# The transfer of maternal antibodies and dynamics of maternal and natural infection-induced antibodies against coxsackievirus A16 in Chinese children 0–13 years of age: a longitudinal cohort study

**DOI:** 10.1186/s12916-022-02604-w

**Published:** 2022-11-09

**Authors:** Jiaxin Zhou, Yonghong Zhou, Kaiwei Luo, Qiaohong Liao, Wen Zheng, Hui Gong, Huilin Shi, Shanlu Zhao, Kai Wang, Qi Qiu, Bingbing Dai, Lingshuang Ren, Lili Wang, Lidong Gao, Meng Xu, Nuolan Liu, Wanying Lu, Nan Zheng, Xinhua Chen, Zhiyuan Chen, Juan Yang, Simon Cauchemez, Hongjie Yu

**Affiliations:** 1 School of Public Health, Fudan University, Key Laboratory of Public Health Safety, Ministry of Education, Shanghai, China; 2 Hunan Provincial Center for Disease Control and Prevention, Changsha, China; 3 Anhua County Center for Disease Control and Prevention, Yiyang, China; 4 Mathematical Modelling of Infectious Diseases Unit, Institut Pasteur, Université de Paris, UMR2000, CNRS, 75015 Paris, France

**Keywords:** Coxsackievirus A16 (CVA16), Transplacental transfer, Maternal antibody, Natural infection, Antibody kinetics

## Abstract

**Background:**

A major hand-foot-and-mouth disease (HFMD) pathogen, coxsackievirus A16 (CVA16), has predominated in several of the last 10 years and caused the largest number of HFMD outbreaks between 2011 and 2018 in China. We evaluated the efficacy of maternal anti-CVA16 antibody transfer via the placenta and explored the dynamics of maternal and natural infection-induced neutralizing antibodies in children.

**Methods:**

Two population-based longitudinal cohorts in southern China were studied during 2013–2018. Participants were enrolled in autumn 2013, including 2475 children aged 1–9 years old and 1066 mother-neonate pairs, and followed for 3 years. Blood/cord samples were collected for CVA16-neutralizing antibody detection. The maternal antibody transfer efficacy, age-specific seroprevalence, geometric mean titre (GMT) and immune response kinetics were estimated.

**Results:**

The average maternal antibody transfer ratio was 0.88 (95% CI 0.80–0.96). Transferred maternal antibody levels declined rapidly (half-life: 2.0 months, 95% CI 1.9–2.2 months). The GMT decayed below the positive threshold (8) by 1.5 months of age. Due to natural infections, it increased above 8 after 1.4 years and reached 32 by 5 years of age, thereafter dropping slightly. Although the average duration of maternal antibody-mediated protection was < 3 months, the duration extended to 6 months on average for mothers with titres ≥ 64.

**Conclusions:**

Anti-CVA16 maternal antibodies are efficiently transferred to neonates, but their levels decline quickly. Children aged 0–5 years are the main susceptible population and should be protected by CVA16 vaccination, with the optimal vaccination time between 1.5 months and 1 year of age.

**Supplementary Information:**

The online version contains supplementary material available at 10.1186/s12916-022-02604-w.

## Background

Coxsackie A16 (CVA16) belongs to the picornavirus family, which can cause a wide spectrum of diseases, such as encephalitis, paralysis or myelitis, meningitis, hand-foot-and-mouth disease (HFMD), upper and lower respiratory tract diseases, pleurodynia, herpangina, and myopericarditis [1]. HFMD is a common infectious disease, with CVA16 as the major pathogen, mainly observed among children under 5 years old in the Asia-Pacific region, especially in China. CVA16 predominated in China in 2012, 2014, 2016 and 2018, accounting for 58.6% of HFMD outbreaks between 2011 and 2018. An increasing number of HFMD cases caused by CVA16 have been observed since 2017 [2]. Although most patients show minor symptoms, CVA16 can cause severe and fatal HFMD, and increasing numbers of reports of such outcomes have been noted in the US, France, Japan, mainland China, and Taiwan of China [3]. In Shenyang, northeastern China, 20.7% (19/92) of HFMD cases with neurological symptoms were caused by CVA16 infection, of which 2 patients had brainstem encephalitis and 1 had acute flaccid paralysis [4].

There is currently no specific treatment for HFMD, and the CVA16 HFMD vaccine is still in the preclinical phase, with promising results in animal models [3]. The only HFMD vaccine licenced in mainland China is the enterovirus A71 (EV-A71) monovalent HFMD vaccine, but this vaccine fails to confer cross-protection against HFMD caused by CVA16 or other enteroviruses [5–7]. All of these elements highlight the urgent need for the development of vaccines targeting CVA16.

Maternal antibodies have been recognized as an effective source to protect newborns against measles virus and influenza [8, 9]. Given the absence of a CVA16 vaccine, anti-CVA16 maternal antibodies, as the only source of passive immunity, may play an important role against CVA16 during the early months of life. After the waning of maternal antibodies, young children may become susceptible to infection. Serological studies are necessary to quantify maternal antibody transfer and anti-CVA16 antibody dynamics, including maternally transmitted antibodies as well as natural infection-induced antibodies. Such understanding may help select the target population for future CVA16 vaccination campaigns. Few studies have reported a positive correlation of anti-CVA16 antibody levels in mother-neonate pairs [10–13]. Only one study reported the average transfer ratio of anti-CVA16 maternal antibodies [11], and the half-life of maternal antibodies was not reported. Limited cohort studies describing maternal antibody dynamics showed that seroprevalence in neonates decreased from 72% at birth to 0–26.4% at 6–7 months of age. However, these results may be affected by the small sample size [12] and limited follow-up [14, 15]. In addition, previous studies were cross-sectional studies, which cannot reveal the dynamics of natural infection-induced antibodies over time [16–20].

Here, we quantify the transfer of maternal antibodies and the dynamics of antibodies from birth to 13 years old using two population-based cohorts: one was a paired mother-neonate cohort (abbr. neonate cohort), and the other was a 1- to 9-year-old paediatric cohort (abbr. paediatric cohort). More specifically, we quantified concentrations of CVA16-specific antibodies in paired maternal and cord serum samples and assessed the transplacental transfer efficiency of maternal antibodies from a large cohort of mothers and neonates. We then analysed the antibody kinetics from birth to age 13 years (including the decline in maternal antibody levels and subsequent increases due to natural infection) using the paediatric cohort and neonate cohort together.

## Methods

### Study design and participants

**Fig. 1:**
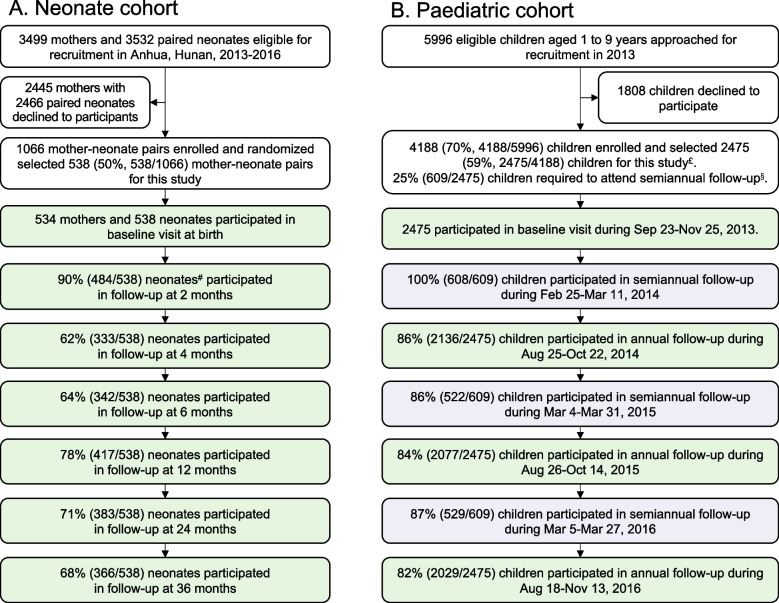
Recruitment and follow-up rates for the two cohorts. ^**#**^Residual serum samples from 538 participants were available at baseline, while quantities of sera from one participant at 2 months of age were insufficient for the CVA16 neutralization test. ^£^Details of the selection are described in the appendix (Additional file 1: Table S1). ^§^A subgroup of children (25%, 609/2475) in the paediatric cohort attended semiannual follow-up in addition to the routine annual follow-up

Two population-based longitudinal cohorts were established in three townships (Tianzhuang, Jiangnan, and Qingtang) in Anhua County, Hunan Province, a rural area in southern China, including a paired mother-neonate cohort and a 1- to 9-year-old paediatric cohort. The neonate cohort design has been described previously [21]. Briefly, neonates who were born after Sep 20, 2013, and whose mothers resided in the area for at least 3 months were eligible for the mother-neonate paired cohort [21]. A total of 1066 pairs of mothers-neonates out of 3499 eligible mothers and 3532 eligible neonates were enrolled from Sep 20, 2013, to Oct 14, 2015, and we randomly selected half of these participants for this study (Fig. 1A). For the paediatric cohort, children who were 1–9 years old of age at enrolment from Sep to Nov 2013 and who resided in the study sites for more than 3 months were recruited. The 1-year age group was defined as those children aged 12–23 months; other age groups were defined in a similar way. Stratified random sampling of three townships was conducted from the eligible list to select children in each age group. Ultimately, 4188 children aged 1–9 years were enrolled out of 5996 eligible children, and 2475 children were selected to join this study (Fig. 1B). The details of the selection method are described in Additional file 1: Table S1. In addition, enhanced virological monitoring of HFMD was carried out locally in Anhua County from 2013 to 2016 [22].

### Procedures

Taking the village as the unit, the parents led the children to the township health centre within the study period to participate in the research. If the participants were unable or had no time to go to the township health centre, the well-trained project personnel and village doctors investigated the children by household survey as an auxiliary method. For the neonate cohort, a venous blood sample (2 ml) from mothers was collected around the time of delivery, and a cord blood sample (2 ml) was collected for each neonate at baseline. Mothers were invited to answer a questionnaire about basic demographic information and delivery information (such as gestational weeks, delivery method, and neonatal birthweight). After that, the neonates were followed up at 2, 4, 6, 12, 24, and 36 months. Details about the procedures of the neonate cohort can be found elsewhere [21].

For the paediatric cohort, blood samples were collected at baseline during Sep–Nov 2013. Each child was invited to attend the annual follow-up visit between August and November during 2014–2016 for a total of 3 annual follow-ups. A subset of children was invited to attend another semiannual follow-up visit between February and March during 2014–2016 (total of 6 follow-ups). For both the neonatal and paediatric cohorts, blood samples (2 ml) and questionnaires about hygiene habits and vaccination history were collected at each follow-up by well-trained nurses and epidemiological investigators, respectively.

CVA16 activity was monitored through enhanced virological surveillance of HFMD in Anhua [22] and national notifiable disease surveillance data in Hunan [23].

### Laboratory procedures

We performed a neutralization assay to measure serum neutralizing antibodies as previously described [24]. In brief, serum dilutions (ranging from 1:8 to 1:1024) were mixed with an equal volume of 100 TCID50 of the virus strain, with a 2-fold serial dilution. All diluted samples were tested in duplicate, and the titration was double-checked. Antibody titres were defined as the reciprocal of the highest dilution capable of inhibiting 50% of the cytopathic effect and calculated by use of the Karber method [25] (Additional file 1: page 9, line 43–56).

### Statistical analysis

The comparison of baseline demographic information between the two groups was conducted with the chi-square test for discrete variables and the Wilcoxon test for continuous variables. An antibody titre ≥ 8 was chosen as the positivity threshold; we considered 16 to indicate positivity in a sensitivity analysis. The antibody titre was log2-transformed before analysis. Seroprevalence and the geometric mean titre (GMT) were calculated to estimate the maternal and neonatal immunity levels. The average transfer ratio of maternal antibody was defined as the geometric mean of the neonate-to-mother ratio, conditional on positivity of the mother [21]. All mother-neonate pairs (i.e., including those with seronegative mothers) were assessed in a sensitivity analysis.

Generalized linear regression was conducted to identify factors associated with neonatal titre, neonatal seropositivity and the transfer ratio. A generalized linear mixed model with a B-spline was used to estimate the kinetics of the immune response over age. The antibody dynamics specified by three maternal antibody levels (negative group: <8, low level: 8–<64, and high level: 64–512) were described to explore the effect of maternal antibody level on the children’s antibody dynamics, with the range of maternal antibody being 6 to 512. The Kaplan–Meier method was conducted to estimate the median time to losing protective maternal antibodies, and linear regression analysis was used to estimate the relationship between the duration of protection and maternal antibody titres. Survival analysis considering interval censoring and interval regression were conducted as a sensitivity analysis. All analyses were performed in R (version 4.0.2) and SAS (version 9.4).

## Results

A total of 3013 children, including 538 neonates and 2475 children aged 1–9 years old, were enrolled in our study between Sep 2013 and Sep 2018. The follow-up rate of the neonate cohort was above 60% (Fig. 1A). Over 80% of children in the paediatric cohort were followed up annually. The group with semiannual follow-up had at least an 85% follow-up rate from Sep 2013 to Sep 2016 (Fig. 1B). The local circulation of CVA16 during 2013–2018 in Anhua County, Hunan Province, is depicted in the Appendix (Additional file 1: page 6–8, line 6–42).

The general characteristics of the enrolled neonates and children at baseline are provided in Table 1. The median age of the participants was 3 years old (IQR: 1–6), and 1532 (51%) children were boys. The follow-up time ranged from 1 to 41 months (median: 22 months; IQR: 11–29 months). The median age of mothers at baseline was 24 years old (IQR: 26–29 years old). Ninety percent of the mothers’ sera were collected at delivery (Additional file 1: Table S4 (a)). Comparisons between participants and local nonparticipants are presented in the appendix (Additional file 1: pages 12–16, line 118–125).

**Table 1:** Participant demographic characteristics at baseline

Characteristics	Neonates (***n***=538)	1–9-year-old children (***n***=2475)	Total (***n***=3013)
Age at baseline (years)			
Median (IQR)	-	4 (2-6)	3 (1–6)
0	538 (100)	-	538 (18)
1	-	355 (14)	355 (12)
2	-	368 (15)	368 (12)
3	-	332 (13)	332 (11)
4	-	333 (13)	333 (11)
5	-	330 (13)	330 (11)
6	-	283 (11)	283 (9)
7	-	205 (8)	205 (7)
8	-	162 (7)	162 (5)
9	-	107 (4)	107 (4)
Male	286 (53)	1246 (50)	1532 (51)
Has twin siblings^a^	12 (2%)	-	12
Gestational age at birth (weeks)			
Median (IQR)	40 (39-41)	-	-
<37	10 (2)	105 (4)	115 (4)
37–42	508 (94)	2344 (95)	2852 (95)
>42	20 (4)	25 (1)	45 (1)
Delivery mode			
Transvaginal delivery	331 (62)	1577 (64)	1908 (63)
Caesarean	207 (38)	897 (36)	1104 (37)
Birthweight (g)			
Median (IQR)	3300 (3000–3600)	3250 (3000–3500)	3250 (3000–3500)
<2500	11 (2)	88 (4)	99 (3)
2500–<4000	491 (91)	2139 (86)	2630 (87)
≥4000	36 (7)	247 (10)	283 (9)
Annual family income (RMB, Yuan)			
<20,000	82 (15)	611 (25)	693 (23)
20,000–<50,000	297 (55)	1382 (56)	1679 (56)
≥50,000	158 (29)	482 (19)	640 (21)

Data are *n* (%). ^a^4 pairs of twins, and 4 neonates had a twin sibling, but their twin sibling did not participate in our study

**Fig. 2:**
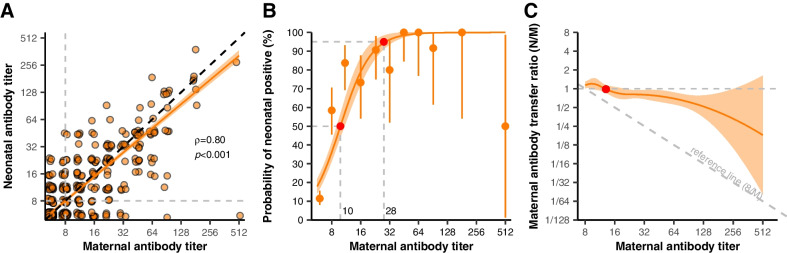
Anti-CVA16 maternal antibody titre transfer efficacy. **A** Correlation of anti-CVA16 antibodies between mother and neonates. **B** The probability for neonatal seropositivity at birth over different maternal antibody levels. The points and vertical bars indicate calculated seropositivity and 95% confidence intervals. Solid lines and shadows indicate predicted average seropositivity and 95% confidence interval bands. **C** The transfer ratio trend over maternal antibody titre. Two grey dashed lines are presented in panel C. The horizontal grey line indicates that mothers and neonates had the same antibody titre, and another grey dashed line indicates that neonates had the minimum positive maternal antibody titre (8)

As shown in Fig. 2A, the baseline anti-CVA16 antibody titre of neonates at birth was highly correlated with the maternal anti-CVA16 antibody titre (*ρ*=0.80, 95% CI 0.77–0.83). The GMT and seroprevalence were similar between mothers and neonates at birth (mother *vs.* neonate: 9.82 (95% CI 9.14–10.55) *vs.* 9.61 (95% CI 8.97–10.30), *p*= 0.102; 44.80% (95% CI 40.53–49.10) *vs.* 41.82% (95% CI 37.61–46.12), *p*= 0.349, respectively).

The probability of neonatal positivity increased as the maternal antibody titre rose until the maternal antibody titre was >28 for CVA16, with the neonatal probability being close to 100% (Fig. 2B). The overall maternal anti-CVA16 antibody titre transfer ratio was 0.88 (95% CI 0.80–0.96). It decreased with maternal antibody levels (Fig. 2C), with an average value larger than 1 when maternal antibody titres were ≤13. Although the transfer efficacy decayed, neonates can successively obtain positive maternal antibodies via placental transmission as long as the maternal antibody titre reaches the positive level (Fig. 2C, Additional file 1: Fig. S4).

Multivariate analysis (Additional file 1: Table S8) indicated that maternal anti-CVA16 antibody titres were highly correlated with neonates’ titres at birth and the status of antibody titres (positive or not). For every 2-fold increase in maternal titres, neonatal titres increased by 69.1% (95% CI 63.4–75.2). The probability of acquiring positive antibody levels for neonates at birth increased by 17.7 times (95% CI 15.5–20.0) for every 2-fold increase in maternal titres. Having a twin decreased the probability by 15.7% (95% CI 1.4–28.0) compared to being a single neonate. The transfer ratio decreased by 14.8% (95% CI 11.8–17.6%) for every 2-fold increase in maternal antibody titre. The transfer ratio was reduced by 16.4% (95% CI 2.8–28.1%) when the mother had liver system disease.

Sensitive analyses with a positive threshold at 16 are depicted in the appendix (Additional file 1: Fig. S7).

**Fig. 3:**
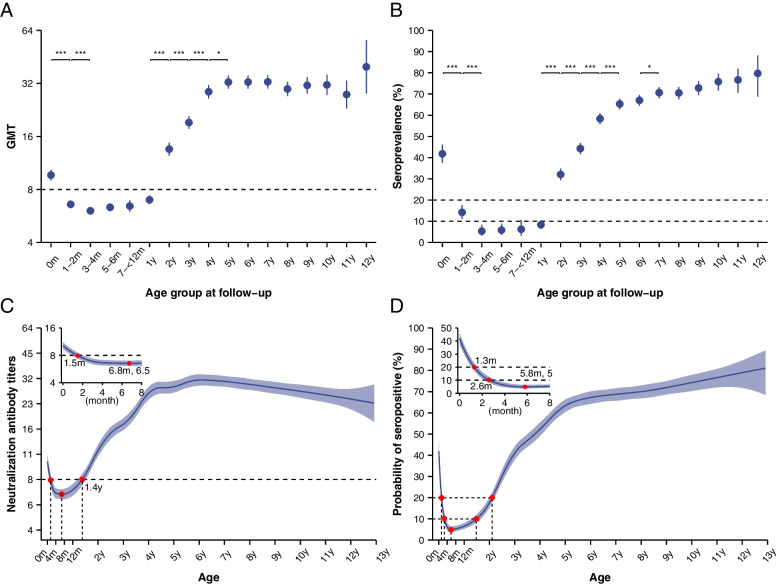
Dynamic pattern of CVA16 neutralization antibody titre and seroprevalence over time. **A** Observed GMT stratified by age. **B** Observed seroprevalence stratified by age. The points and vertical bars indicate the mean and 95% confidence interval. *** and * indicate *p*<0.001 and *p*<0.05, respectively. **C** Fitted anti-CVA16 antibody titre dynamics over age by generalized linear mixed model. **D** Fitted probability of seropositive over age by generalized linear mixed model. The solid line and shadow indicate the fitted mean and 95% confidence band. 1:8 is the positive threshold

Maternal transmitted antibody levels in neonates diminished rapidly. This drop was followed by an increase in titres due to natural infection, as was apparent in both the GMT and seroprevalence stratified by age groups (Fig. 3A, B). Forty-two per cent of neonates had positive antibody levels, with a GMT of 10 (95% CI 9–10). However, the GMT decayed below the positive threshold after 1.5 months (range: 1.0–2.0 months) and further decreased to a minimum at 6.8 months. The seroprevalence experienced a sharp drop, falling to 20% after 1.3 months, with the lowest point at 5.8 months. Then, due to natural infections, the seroprevalence increased rapidly from 6 months to 5 years old (60%), then slowly increased to 80% at 13 years old. The GMT increased above the positive threshold after 1.4 years (range: 1.2–1.5 years) and then reached 32 by the age of 5 years. It slightly dropped thereafter. For the sensitivity analysis with a higher threshold of 16, the kinetics of seroprevalence and the GMT were consistent with those in the baseline analysis. The GMT increased above 16 by 2.7 (range: 2.4–3.1) years of age (Additional file 1: Fig. S7). The kinetics of antibodies from high-level, low-level and negative maternal antibody groups were significantly different within 6 months. The higher the level of maternal antibody, the longer the duration of neonatal titre decay to under the detective threshold (high-level *vs*. low-level: 5.3 months *vs*. 1.2 months) and to the bottom (high-level *vs*. low-level: 11.0 months *vs*. 8.1 months). The antibody dynamics in the lower level of maternal antibody groups, especially the negative group, rose earlier due to natural infection, and the corresponding times for the three groups to rise to 8 were 1.5, 2.0 and 2.1 years, respectively. However, the difference was not obvious when age more than 1.5 years old (Additional file 1: Fig. S9).

**Fig. 4:**
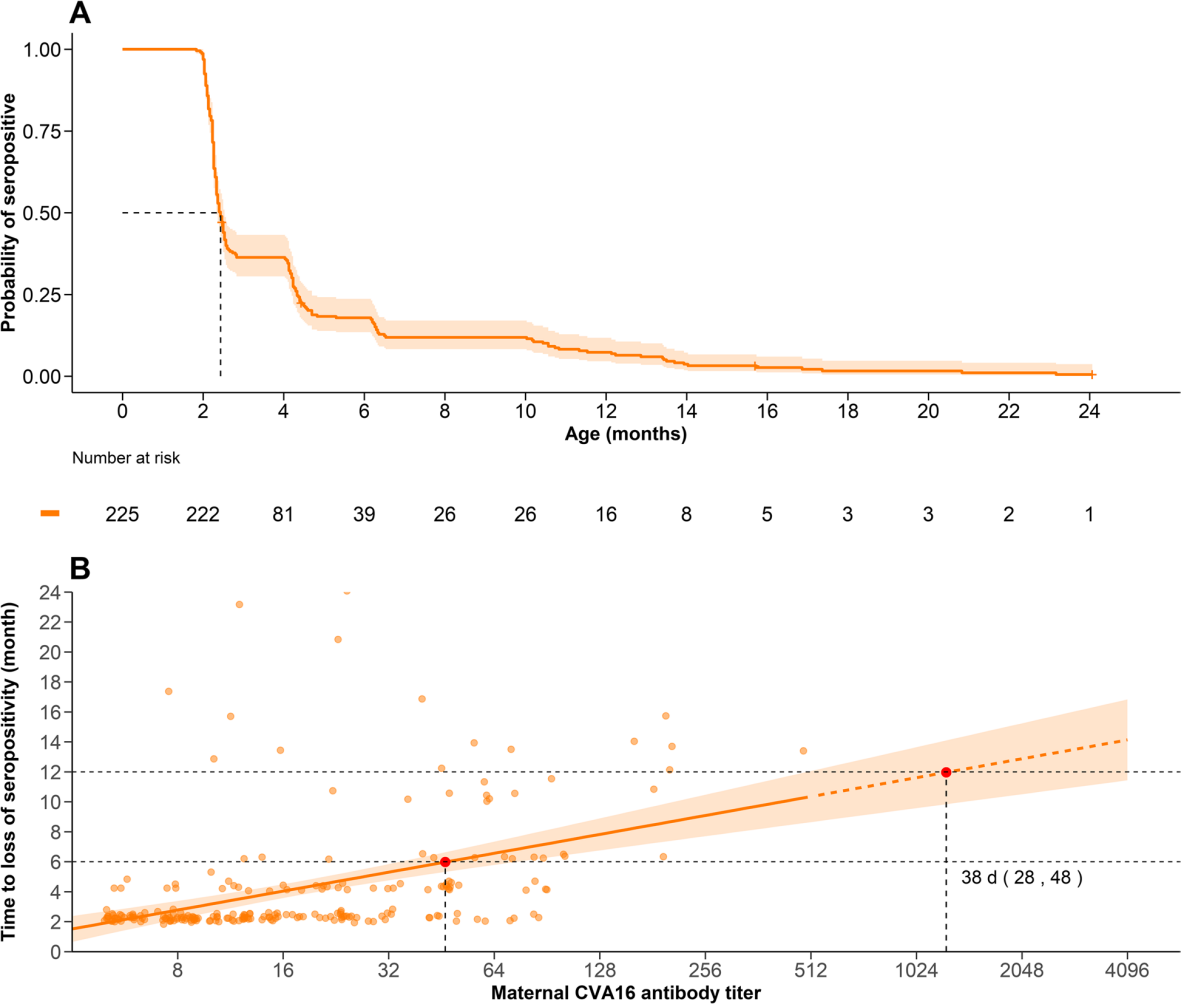
Loss of protective immunity acquired from mothers and the relationship between duration and maternal antibody titre. **A** The Kaplan–Meier survival curve for the probability of seropositivity in neonates who received maternal antibodies at birth. **B** Relationship between time to loss of seropositive immunity and maternal antibody titres

The maternally transmitted antibody levels decayed faster than natural infection-induced antibody titres (*p*<0.001), with corresponding antibody half-lives of 2.0 months (95% CI 1.9–2.2 months) and 13.9 months (95% CI 13.3–14.5 months), respectively (Additional file 1: Fig. S10). Half of the neonates who were seropositive at birth were seronegative by 2.4 months of age (95% CI 2.3–2.5 months) (Fig. 4A). The average time to loss of seropositivity was 2.8 months (95% CI 2.5–3.1 months). For every 2-fold increase in maternal antibody, the duration of protection was prolonged by approximately 38 days (95% CI 28–48 days). When the maternal antibody titre was ≥64 and >512, the duration of protection for neonates was extended to 6 and 12 months, respectively (Fig. 4B). The sensitivity analyses are shown in Additional file 1: Table S13.

## Discussion

Our study revealed that the average anti-CVA16 antibody titres and seroprevalence were similar in maternal and cord blood, with a mean transfer ratio of maternal antibodies of 0.88 (95% CI 0.80–0.96). Although the transfer ratio decreased as the maternal antibody titre increased, the positive maternal antibodies could still be successfully transmitted to neonates. However, due to the low maternal seroprevalence, only 42% of neonates acquired positive concentrations of anti-CVA16 antibodies from mothers, which declined rapidly, with a half-life of 2.0 months (95% CI 1.9–2.2 months). Among them, the average duration of protection from maternal antibodies was 2.8 months (95% CI 2.5–3.1 months), and the duration was extended to 6 months on average for mothers with titres of ≥64. After the disappearance of maternal antibodies, antibody titres increased because of rising natural infections.

Transplacental antibody transfer from mother to foetus provides protection from infection in the first weeks of life, such as measles, rubella, diphtheria, and tetanus, with various transfer efficacy. A strong association between maternal anti-CVA16 antibodies and neonates with a correlation coefficient of 0.8 was observed in our study, which was close to 0.56–0.75 in previous studies [10–12]. The transfer of anti-CVA16 maternal antibody is efficient, with an overall transfer ratio of 0.88, which is similar to Fu’s observation of 0.9 [11] and is lower than that for EV-A71 [21], measles, and poliomyelitis III antibodies [11, 21]. The trend of the transfer ratio decreasing as the maternal antibody titre increases has also been observed for measles [26] and EV-A71 [21], which may be due to the saturation of a finite number of Fc receptors [26, 27]. Maternal antibody level is the major factor associated with the transfer ratio, following the mother’s health. Liver system disease in the mother was associated with a lower transfer ratio. Other factors associated with mothers, such as maternal age, gestational diabetes, gestational hypertension, parity and gravidity, as well as factors associated with neonates, such as gestational age and birth weight, were not found to be related to transfer in our study.

The seroprevalence and GMT in our study were minimal at the ages of 5.8 months and 6.8 months, respectively, in line with previous longitudinal observations [12, 14, 15, 28]. The neonatal seroprevalences at birth and 2-4 months of age were lower than those in the studies by Jiangsu, potentially because a higher prevalence (90%) was observed in mothers in those studies [15, 29]. The time when the GMT reached 8 and when it reached its minimum value were similar to those in Guangzhou studies (at 3 and 6 months old) [12, 28]. No previous longitudinal study investigated the kinetics of the anti-CVA16 antibody level, and only cross-sectional studies reported age-specific seroprevalence and GMT. Our age-specific seroprevalence results between 6 months and 13 years were comparable with those of previous reports in China [30], Singapore [17], Korea [16] and Thailand [18].

Using a neonatal cohort, we showed in another study that the dynamics of anti-EV-A71 antibodies had a similar pattern [21]. With higher maternal seroprevalence, neonatal EV-A71 seroprevalence was significantly higher than that of CVA16 for neonates aged 0–4 months old, so it took slightly longer for the GMT to decay below the positive threshold. After the decay of maternal antibody, the seroprevalence was similar between CVA16 and EV-A71 within 4 months to 3-year-old children (unpublished study), although the EV-A71 GMT was higher than the CVA16 GMT. A similar pattern was also observed in previous studies [31–33], showing that EV-A71 might elicit stronger neutralizing antibody responses than CVA16. The capsid proteins of the two viruses have approximately 20% sequence differences, as well as antigenic variation caused by the different surface structures of the two viruses, which may explain this phenomenon [34].

After the rapid decay of maternal antibodies, the seroprevalence increased with age as a result of natural infection; however, 40–95% of children from 6 months to 5 years old were still seronegative. In addition, to curb the transmission of CVA16 infection, a threshold of 59–96% for herd immunity is needed [2, 35–37]. Meanwhile, 0- to 5-year-old children, accounting for 99% and 96.5% of severe and mild cases in Hunan Province, respectively, constitute the majority of HFMD cases associated with CVA16 (Additional file 1: Fig. S11). Among them, the 1-year-old group had the largest number of mild and severe cases, which was in line with a previous report [23]. The above evidence showed that children aged 0–5 years old represent the main susceptible population, which should be targeted for vaccination.

Overall, both avoiding potential maternal antibody interference and preventing the largest incidence, especially severe cases, in the 1-year-old group, we recommend that the optimal vaccination time be between 1.5 months and 1 year old. Complete vaccination during this period could prolong the positive antibody titre until 5 years old, although this hypothesis is based on the half-life of anti-CVA16 antibody induced by infection. Further research is required on antibody dynamics induced by vaccines. Thus, we call for accelerating the development of a human CVA16 vaccine and recommend clinical trials to further assess doses, safety, and effectiveness in susceptible young children aged 1.5 months to 5 years old.

Vaccinating pregnant mothers to provide maternal antibodies to infants who are too young to be vaccinated could be an alternative intervention to protect these infants, which has proven efficacious for influenza, pertussis and COVID-19 [8, 38, 39]. As we estimated, anti-CVA16 maternal antibodies can be effectively transmitted to newborns, and high maternal antibody levels can prolong the duration of protection for infants, with protection lasting up to 6 months when the maternal antibody level is ≥ 64. Meanwhile, the results of antibody dynamics specified by maternal antibody levels also indicated that a higher maternal antibody group was associated with later antibody decay to undetectable levels and later natural infection. It is possible to increase the mother’s antibody titre to extend protection to 1 year of age. Given that, vaccination of mothers during pregnancy can be a solution to protect infants from disease and death in early life, especially those under 1 year old. This conclusion is based on observations of antibodies mounted upon natural infection rather than vaccination, and we therefore still require further research to elucidate the efficacy or effectiveness of maternal CVA16 vaccination, antibody response and duration of immunity produced by maternal CVA16 vaccination.

A study in Vietnam found that adults can have HFMD and contribute to household transmission [40]. In our study, the seroprevalence of mothers (44.8%) was lower than that of mothers (87–93%) and adults (65–83%) in some coastal provinces of China [11, 15], Germany [19] and Vietnam [40]. Given the absence of sex-specific and age-specific differences in CVA16 prevalence [19, 41, 42], males and adults of other ages are also susceptible in theory. Thus, vaccination for adults in close contact with infants could help reduce household transmission and provide indirect protection for vulnerable infants.

This study is a population-based longitudinal study with a large sample size, prolonged follow-up design and high follow-up rates, in contrast with those of previous studies [10, 11, 14–17]. The quality control of the neutralization test was stringent. The conclusion using children with complete follow-up is consistent with the main results (Additional file 1: Fig. S8). However, our study still had some limitations. First, we were unable to correlate antibody levels with clinical symptoms because only a few cases were reported during the follow-up period. Moreover, there are currently no studies confirming the association between antibody titre and symptom reduction or avoidance of infection. Thus, the positive threshold in this study is a threshold for detecting antibodies in neutralization assays. It is expected that a higher titre is associated with higher protection; thus, a sensitivity analysis with 16 as the positive threshold was conducted, which showed a conclusion consistent with that of the baseline analysis. Second, we were unable to compare the demographic information between the study participants and the local eligible nonenrollees in the 1–9 years old cohort because they refused to provide personal information. Finally, the sample size of mothers with higher antibodies (≥256) in our study was relatively small, causing the relative decreasing trend of directly calculated prevalence when the maternal antibody titre was 512, while the predicted probability was stable due to logit transformation in logistic regression.

## Conclusions

In conclusion, positive titres of anti-CVA16 antibodies were efficiently transferred to neonates from mothers through the placenta. However, titres declined rapidly to below the positive threshold by 1.5 months of age. Increasing the maternal antibody titre can effectively prolong the duration of protection. The majority of children aged 0–5 years old are susceptible, especially 1-year-old children with a high risk of developing severe disease. It is important to assess the feasibility of vaccinating children between 1.5 months and 1 year old, pregnant women or adults in close contact with infants to protect 0–5-year-old children.

## Supplementary Information

**Additional file 1: Fig. S1.** Time series and predicting epidemic magnitude of CVA16 activity in Anhua county during 2013-2016 (A) and that in Hunan province during 2009-2018 (B). **Fig. S2.** Participants’ included time in baseline and follow-up times (A) and the epidemic seasons of CVA16 activity during 2009-2018 in Anhua, Hunan province (B). **Fig. S3.** Distribution of maternal antibody titre transfer ratio against CVA16 for all pairs of neonate-mother (A) and positive mothers with antibody titre ≥8 (B). **Fig. S4.** The aggregated maternal titre specified transfer ratio. **Fig. S5.** Maternal antibody titre transfers efficacy against CVA16. **Fig. S6.** Transfer ratio trend with all pairs of mother-neonate. **Fig. S7.** Dynamic of seroprevalence, neutralization antibody titre by age for neonates using 16 as positive threshold. **Fig. S8.** Dynamic of neutralization antibody titre by age for neonates using full follow-up subset. **Fig. S9.** Dynamics of neutralization antibody titer by age for neonates stratified by maternal antibody levels. **Fig. S10.** Waning rate of maternal transmitted CVA16 antibody and natural infection induced antibody. **Fig. S11.** Age distribution and cumulative distribution of HFMD associated to CVA16 by clinical severity in Hunan province during 2009-2018 and Anhua county during 2013-2016. **Table S1.** Distribution of participants aged 1-9 years and sampling ratio. **Table S2.** Seasonality comparison between Anhua county and Hunan province. **Table S3.** Epidemic duration for CVA16 circulation in Anhua. **Table S4.** (a) Baseline characteristics of mothers and neonates in neonate cohort between participants and non-participants, selected participants and non-selected participants for CVA16 assay. (b) Baseline characteristics of neonates in neonate cohort between full follow-up participants and incomplete follow-up participants. **Table S5.** Comparison of baseline characteristics of 1-9 years old children between participants and non-selected objects through sampling, full follow-up and incomplete follow-up. **Table S6.** Seroprevalence and GMT of mothers and neonates at birth. **Table S7.** Sample size (n, %) for CVA16 antibody transfer ratio analysis. **Table S8.** Multivariate analysis with factors associated with neonates’ seropositivity, antibody titre at birth and transfer ratio. **Table S9.** Univariate analysis with factors associated with neonates’ seropositivity at birth. **Table S10.** Univariate analysis with factors associated with the transfer ratio of maternal antibody. **Table S11.** Univariate analysis with factors associated with neonates’ antibody titre at birth. **Table S12.** Sensitive analysis for multivariate analysis with factors associated with maternal antibody transfer ratio. **Table S13.** Sensitive analysis of median age of neonate with positive antibody. **Appendix**.

## Data Availability

Data described in the manuscript are available on request due to privacy/ethical restrictions. The analytical code will be made available only to the investigators who have participated in/contributed to the study. The study executive will consider specific requests for data analyses by non-contributing individuals upon reasonable request to the corresponding author.

## Abbreviations

CVA16: Coxsackievirus A16
EV-A71: Enterovirus A71
GMT: Geometric mean titre
Neonate cohort: A paired mother-neonate cohort
Paediatric cohort: A 1- to 9-year-old paediatric cohort
