# Supplementary material for: The transfer of maternal antibodies and dynamics of maternal and natural infection-induced antibodies against coxsackievirus A16 in Chinese children 0–13 years of age: a longitudinal cohort study
Source: BMC Med. 2022 Nov 9;20:436. doi: 10.1186/s12916-022-02604-w (PMC9645321; doi:10.1186/s12916-022-02604-w)
Supplement: Supplementary file 1 — Additional file 1: Fig. S1. Time series and predicting epidemic magnitude of CVA16 activity in Anhua county during 2013-2016 (A) and that in Hunan province during 2009-2018 (B). Fig. S2. Participants’ included time in baseline and follow-up times (A) and the epidemic seasons of CVA16 activity during 2009-2018 in Anhua, Hunan province (B). Fig. S3. Distribution of maternal antibody titre transfer ratio against CVA16 for all pairs of neonate-mother (A) and positive mothers with antibody titre ≥8 (B). Fig. S4. The aggregated maternal titre specified transfer ratio. Fig. S5. Maternal antibody titre transfers efficacy against CVA16. Fig. S6. Transfer ratio trend with all pairs of mother-neonate. Fig. S7. Dynamic of seroprevalence, neutralization antibody titre by age for neonates using 16 as positive threshold. Fig. S8. Dynamic of neutralization antibody titre by age for neonates using full follow-up subset. Fig. S9. Dynamics of neutralization antibody titer by age for neonates stratified by maternal antibody levels. Fig. S10. Waning rate of maternal transmitted CVA16 antibody and natural infection induced antibody. Fig. S11. Age distribution and cumulative distribution of HFMD associated to CVA16 by clinical severity in Hunan province during 2009-2018 and Anhua county during 2013-2016. Table S1. Distribution of participants aged 1-9 years and sampling ratio. Table S2. Seasonality comparison between Anhua county and Hunan province. Table S3. Epidemic duration for CVA16 circulation in Anhua. Table S4. (a) Baseline characteristics of mothers and neonates in neonate cohort between participants and non-participants, selected participants and non-selected participants for CVA16 assay. (b) Baseline characteristics of neonates in neonate cohort between full follow-up participants and incomplete follow-up participants. Table S5. Comparison of baseline characteristics of 1-9 years old children between participants and non-selected objects through sampling, full follow-up and inco [file 12916_2022_2604_MOESM1_ESM.docx]

**Contents**

[Supplementary methods 3](#_Toc115988760)

[1. Sampling method of children from 1-9 years old cohort 3](#_Toc115988761)

[Table S1. Distribution of participants aged 1-9 years and sampling ratio 3](#_Toc115988762)

[2. Time series of CVA16 epidemics during 2009-2018 in Anhua county and Hunan province 6](#_Toc115988763)

[Fig. S1. Time series and predicting epidemic magnitude of CVA16 activity in Anhua county during 2013-2016 (A) and that in Hunan province during 2009-2018 (B). 7](#_Toc115988764)

[Table S2. Seasonality comparison between Anhua county and Hunan province 7](#_Toc115988765)

[Table S3. Epidemic duration for CVA16 circulation in Anhua. 7](#_Toc115988766)

[3. Laboratory procedures 9](#_Toc115988767)

[4. Statistics analysis 9](#_Toc115988768)

[Supplementary results 11](#_Toc115988769)

[1. Participant’s characteristics 11](#_Toc115988770)

[Table S4 (a). Baseline characteristics of mothers and neonates in neonate cohort between participants and non-participants, selected participants and non-selected participants for CVA16 assay. 12](#_Toc115988771)

[Table S4 (b). Baseline characteristics of neonates in neonate cohort between full follow-up participants and incomplete follow-up participants. 13](#_Toc115988772)

[Table S5. Comparison of baseline characteristics of 1-9 years old children between participants and non-selected objects through sampling, full follow-up and incomplete follow-up. 15](#_Toc115988773)

[2. Maternal transfer 17](#_Toc115988774)

[2.1 Maternal antibody titre transfer 17](#_Toc115988775)

[Fig. S3. Distribution of maternal antibody titre transfer ratio against CVA16 for all pairs of neonate-mother (A) and positive mothers with antibody titre ≥8 (B). 17](#_Toc115988776)

[Fig. S4. The aggregated maternal titre specified transfer ratio. 18](#_Toc115988777)

[Fig. S5. Maternal antibody titre transfers efficacy against CVA16. The probability for neonatal positivity over different maternal antibody level (A) and the transfer ratio trend over maternal antibody titre (B) and the aggregated maternal titre specified transfer ratio (C). Grey dashed lines in panel B, and C represented 100% seroprevalence with 16 as threshold, neonates got the titre ≥ 16, and the same transfer ratio, respectively. One side paired t test was used to test the difference between observed transfer ratio and the reference group. 18](#_Toc115988778)

[Table S7. Sample size (n, %) for CVA16 antibody transfer ratio analysis. 19](#_Toc115988779)

[Fig. S6. Transfer ratio trend with all pairs of mother-neonate. 19](#_Toc115988780)

[2.2 Maternal transfer ratio associated factors 19](#_Toc115988781)

[Table S8. Multivariate analysis with factors associated with neonates’ seropositivity, antibody titre at birth and transfer ratio. 19](#_Toc115988782)

[Table S9. Univariate analysis with factors associated with neonates’ seropositivity at birth. 20](#_Toc115988783)

[Table S10. Univariate analysis with factors associated with the transfer ratio of maternal antibody. 22](#_Toc115988784)

[Table S11. Univariate analysis with factors associated with neonates’ antibody titre at birth. 23](#_Toc115988785)

[Table S12. Sensitive analysis for multivariate analysis with factors associated with maternal antibody transfer ratio. 24](#_Toc115988786)

[3. 0-13 years old children CVA16 antibody dynamic 26](#_Toc115988787)

[Fig. S7. Dynamic of seroprevalence, neutralization antibody titre by age for neonates using 16 as positive threshold. 27](#_Toc115988788)

[Fig. S8. Dynamic of neutralization antibody titre by age for neonates using full follow-up subset. 28](#_Toc115988789)

[Fig. S9. Dynamics of neutralization antibody titer by age for neonates stratified by maternal antibody levels. 29](#_Toc115988790)

[4. Decay rate/half-life and time to reduction to negative 29](#_Toc115988791)

[Fig. S10: Waning rate of maternal transmitted CVA16 antibody and natural infection induced antibody. 30](#_Toc115988792)

[Table S13. Sensitive analysis of median age of neonate with positive antibody. 30](#_Toc115988793)

[5. Disease burden of HFMD related to CVA16 30](#_Toc115988794)

[Fig. S11. Age distribution and cumulative distribution of HFMD associated to CVA16 by clinical severity in Hunan province during 2009-2018 and Anhua county during 2013-2016. 31](#_Toc115988795)

# Supplementary methods

# Sampling method of children from 1-9 years old cohort

### Table S1. Distribution of participants aged 1-9 years and sampling ratio

|  | **No. of follow-up visits participated** | **No. participants and sampling for lab-test on CVA16 antibody** | **Age group** | | | | | | **Total** |
| --- | --- | --- | --- | --- | --- | --- | --- | --- | --- |
|  |  |  | **1-year** | **2-year** | **3-year** | **4-year** | **5-year** | **6-9 year** |  |
| Those who participated to annual follow-up visits | 1 | No. all participants | 40 | 49 | 28 | 29 | 25 | 26 | 197 |
|  |  | No. participants with lab-test performed | 20 | 24 | 14 | 15 | 13 | 26 | 112 |
|  |  | Sampling ratio* | 50% | 49% | 50% | 52% | 52% | 100% | 57% |
|  | 2 | No. all participants | 61 | 47 | 34 | 43 | 36 | 44 | 265 |
|  |  | No. participants with lab-test performed | 31 | 27 | 18 | 22 | 18 | 44 | 160 |
|  |  | Sampling ratio* | 50% | 57% | 53% | 51% | 50% | 100% | 61% |
|  | 3 | No. all participants | 88 | 100 | 65 | 82 | 84 | 115 | 534 |
|  |  | No. participants with lab-test performed | 45 | 49 | 34 | 41 | 42 | 115 | 326 |
|  |  | Sampling ratio* | 51% | 49% | 52% | 50% | 50% | 100% | 61% |
|  | 4 | No. all participants | 343 | 368 | 356 | 339 | 343 | 396 | 2145 |
|  |  | No. participants with lab-test performed | 173 | 182 | 179 | 169 | 169 | 396 | 1268 |
|  |  | Sampling ratio* | 50% | 49% | 50% | 50% | 49% | 100% | 59% |
| Subgroup who participated to semi-annual follow-up visits | 2 | No. all participants | 7 | 3 | 2 | 2 | 5 | 5 | 24 |
|  |  | No. participants with lab-test performed | 3 | 2 | 2 | 1 | 3 | 5 | 16 |
|  |  | Sampling ratio* | 43% | 67% | 100% | 50% | 60% | 100% | 67% |
|  | 3 | No. all participants | 10 | 5 | 5 | 7 | 2 | 2 | 31 |
|  |  | No. participants with lab-test performed | 5 | 2 | 3 | 4 | 1 | 2 | 17 |
|  |  | Sampling ratio* | 50% | 40% | 60% | 57% | 50% | 100% | 55% |
|  | 4 | No. all participants | 11 | 9 | 4 | 5 | 5 | 3 | 37 |
|  |  | No. participants with lab-test performed | 5 | 4 | 2 | 3 | 3 | 3 | 20 |
|  |  | Sampling ratio* | 45% | 44% | 50% | 60% | 60% | 100% | 54% |
|  | 5 | No. all participants | 10 | 16 | 13 | 11 | 11 | 10 | 71 |
|  |  | No. participants with lab-test performed | 5 | 9 | 7 | 6 | 5 | 10 | 42 |
|  |  | Sampling ratio* | 50% | 56% | 54% | 55% | 45% | 100% | 59% |
|  | 6 | No. all participants | 25 | 29 | 31 | 17 | 17 | 19 | 138 |
|  |  | No. participants with lab-test performed | 13 | 14 | 16 | 9 | 8 | 19 | 79 |
|  |  | Sampling ratio* | 52% | 48% | 52% | 53% | 47% | 100% | 57% |
|  | 7 | No. all participants | 114 | 115 | 114 | 129 | 137 | 137 | 746 |
|  |  | No. participants with lab-test performed | 55 | 55 | 57 | 63 | 68 | 137 | 435 |
|  |  | Sampling ratio* | 48% | 48% | 50% | 49% | 50% | 100% | 58% |
| Total | | No. all participants | 709 | 741 | 652 | 664 | 665 | 757 | 4188 |
|  |  | No. participants with lab-test performed | 355 | 368 | 332 | 333 | 330 | 757 | 2475 |
|  |  | Sampling ratio* | 50% | 50% | 51% | 50% | 50% | 100% | 59% |

*Sampling ratio=no. of those with lab-test performed/no. of all participants in each stratification.

# Time series of CVA16 epidemics during 2009-2018 in Anhua county and Hunan province

We used two datasets of HFMD surveillance to characterize the CVA16 circulation in Hunan province especially Anhua county. One is national enhanced HFMD surveillance in Hunan province during Oct 2009 to Oct 2018. Except the national HFMD surveillance in Hunan province, we conducted a hospital-based enhanced HFMD surveillance at six hospitals in Anhua county, Hunan province during Oct 2013 to Sep 2016. All HFMD inpatients aged 14 years old were sampled and tested for defining the infection as well as serotype causing infection using RT-PCR and nested RT-PCR[22].

We standardized the weekly positive cases by the total number of detective cases in corresponding year to adjust the variety of annual detected cases’ number prior to comparing the seasonality of CVA16 epidemic in Anhua county with that in Hunan province during the common sampling period (Oct 2013 to Oct 2016). The “year” was defined as the calendar year since October 1^st^. The time series of CVA16 activity in Anhua county and Hunan province were depicted in Fig. S1. There was highly correlation in standardized CVA16 weekly counts between Anhua county and Hunan province with *ρ*=0.64 (*p*-value < 0.001). We fitted generalized linear regression models to weekly positive cases to obtain quantitative seasonality estimation including annual amplitude, semi-annual amplitude, ratio of periodicity and annual of peak time of CVA16 epidemic, which were comparable in Anhua and Hunan (Fig. S1, Table S2). Given that, the surveillance data in Hunan province during Oct 2016 to Oct 2018 was used as a proxy for Anhua county from Oct 2016 to Oct 2018.

Using 7-day moving average, choosing the 95% quantile as the critical value of onset of an epidemic and 90% as the end of an epidemic, an epidemic began in the week when weekly CVA16 cases exceeded 95 % quantile and that the epidemic ended the week before indices in four successive weeks were lower than 90%. The epidemic seasons per year were described in table S2.


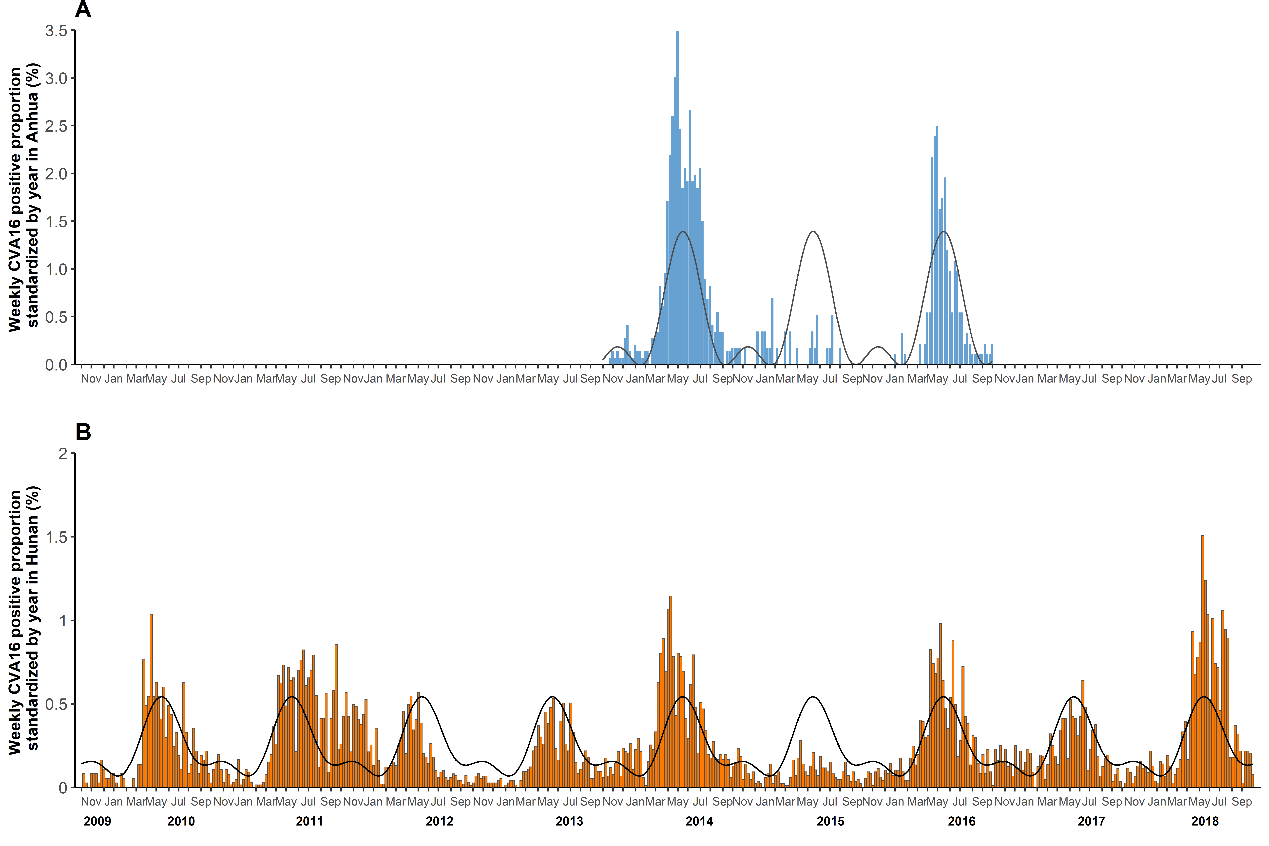


### Fig. S1. Time series and predicting epidemic magnitude of CVA16 activity in Anhua county during 2013-2016 (A) and that in Hunan province during 2009-2018 (B).

### Table S2. Seasonality comparison between Anhua county and Hunan province

|  | **Anhua county**  **(2013.9-2016.10)** | **Hunan province**  **(2009.9-2018.10)** |
| --- | --- | --- |
| Annual amplitude | 0.0060 | 0.0020 |
| Semi-annual amplitude | 0.0033 | 0.0010 |
| Ratio of periodicity | 0.3536 | 0.3334 |
| Annual peak time (week) | 19.18 (April-May) | 20.08 (April-May) |

### Table S3. Epidemic duration for CVA16 circulation in Anhua.

| **Epidemic season** | **Onset of epidemic** | **End of epidemic** | **Duration (weeks)** |
| --- | --- | --- | --- |
| Epidemic1 (2009/10) | 2010/4/5 | 2010/6/7 | 9 |
| Epidemic 2 (2010/11) | 2011/4/25 | 2011/6/27 | 9 |
| Epidemic 3 (2011/12)^#^ | 2011/10/10 | 2011/11/21 | 6 |
| Epidemic 4 (2012/13) | 2013/4/22 | 2013/6/17 | 8 |
| Epidemic 5 (2013/14) | 2014/4/21 | 2014/6/16 | 8 |
| Epidemic 6 (2014/15) | 2014/12/29 | 2015/2/23 | 8 |
| Epidemic 7 (2015/16) | 2016/4/18 | 2016/6/13 | 8 |
| Epidemic 8 (2016/17) | 2017/4/3 | 2017/6/12 | 10 |
| Epidemic 9 (2017/18) | 2018/4/30 | 2018/7/2 | 9 |

^#^The epidemic season during Sep, 2011 to Sep, 2012.


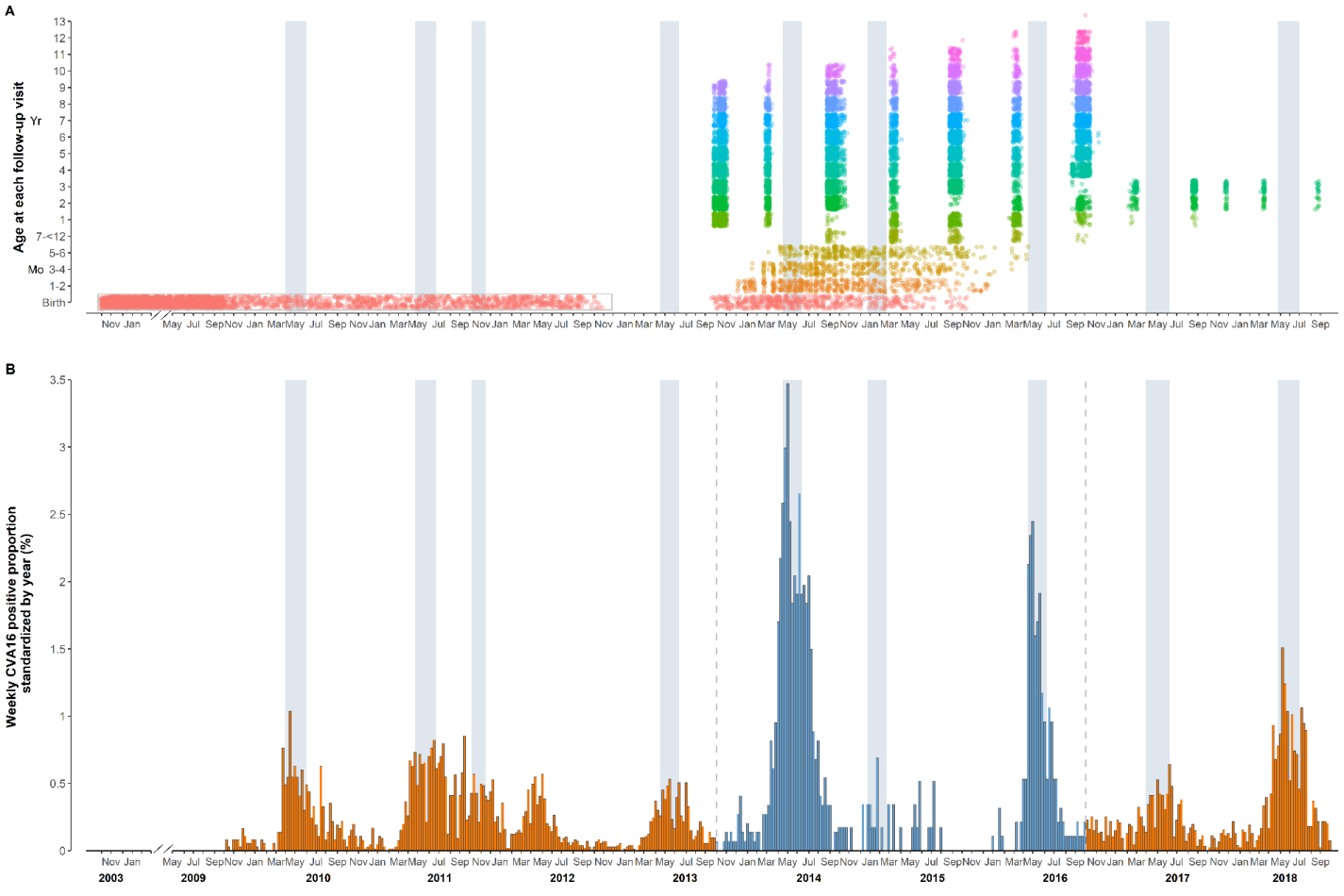


**Fig. S2. Participants’ included time in baseline and follow-up times (A) and the epidemic seasons of CVA16 activity during 2009-2018 in Anhua, Hunan province (B). The observed data during 2013.9 to 2016.9 (the vertical dashed grey line) was based on enhanced surveillance in Anhua county (Blue bar), and the other data was based on national surveillance in Hunan province (Orange bar). Epidemic waves before Sep, 2009 could not be traced given lack of surveillance data. Grey shadows indicate epidemic season.**

# Laboratory procedures

- 1. Virus strains

Virus neutralization test were conducted using the CVA16/190 strain (GenBank accession number: JF420555, genotype B1b) .

- 1. Neutralizing assays

Sera were inactivated at 56°C for 30 min before use, then diluted serially 2-fold from 1:8 to 1:1024 and incubated with equal volume 100 TCID50 virus. After incubation at 37 °C for 2 h, human rhabdomyosarcoma (RD) cells (1~2×105 cells/ml) were added into each well and incubated at 37 °C for 4-7 day. All diluted samples were tested in duplicate. Each reaction plate included a virus control, serum toxicity control and cell control. A virus back titration was performed in each batch of test to determine the amount of virus was within the range of 32-320 TCID50/50μl. Antibody titres were defined as the reciprocal of the highest dilution capable of inhibiting 50% of the cytopathic effect[4] and calculated by use of the Karber method[25]. All titres below 8 were assumed to be 1:6 for calculation.

# Statistics analysis

**Multivariable analysis**

Generalized linear regression was conducted to identify factors associated with neonatal titre, neonatal seropositivity and the transfer ratio.

**Dynamic patterns of antibody titre and probability of positive**

Before analysis, we calculated the ICC between subjects, which was 0.76 showing high correlation within subjects due to the multiple measurement, posing requirement of muti-level model. In our study, a generalized linear mixed model using B-spline was used to identify dynamic patterns of antibody titre and the probability of positive with formula 1 and 2 as models, which considering the autocorrelation within individuals due to repeated measurements. Model selection and the selection of knots for spline were based the minimum AIC.

${log}_{2}{titer}_{ij}=\beta_{0}+b_{0j}+\left( \beta_{1}+b_{1j} \right)\times{s(age}_{ij)}+\varepsilon_{ij}$ (1)

$g\left( P_{ij} \right)=log(\frac{P_{ij}}{1-P_{ij}})=\beta_{0}+b_{0j}+\left( \beta_{1}+b_{1j} \right)\times{s(age}_{ij})+\varepsilon_{ij}$ (2)

Where for visit i, individual j denotes the log-transformed titre for i^th^ visit of infant j, $\beta_{0}$denotes mean intercept, $b_{0j}$denotes individual-specific random intercept, $\beta_{1}$denotes mean slope, $b_{1j}$denotes infant-specific random slope, and $\varepsilon_{ij}$denotes residual error term. S denotes the B-spline formula of age. $P_{ij}$ indicates the probability of positivity for subject j in i^th^ visit.

**Half-life of maternal CVA16 antibodies and natural infection induced antibodies**

To estimate the decay rate of maternal transmitted antibody and the natural infection induced antibody, we checked the individual trajectory of antibody titre. For those neonates with maternal antibody at birth, we excluded the time points when antibody titre was stay negative after maternal antibody decay to negative and when any-fold increased antibody titre and sustained in a relative higher level than before. Similarly, the attenuation of natural infection antibodies started from the highest point of antibodies after infection or previous infections. Infections were defined as

seroconversion (titres moving from below to above the infection cutoff) or at least 4-fold increase between paired serum. Fluctuations within 2-fold during decay trend were attributed to random error.

The same model with formula (1) was used to quantify the half-life (defined as the time at which it would decrease by 50% from baseline) of maternal CVA16 antibody titre and the natural infection induced antibody. Half-life was calculated as $\frac{1}{\beta_{1}}$, and time to loss of protective immunity was estimated by substituting seropositive cutoffs into the equation.

**Survival analysis and the association between maternal antibody titre and positive duration**

For the neonates who successfully got positive antibody, Kaplan–Meier survival curve showing proportion of infants with positive antibody titres at each visit up until 24 months. Infants were censored when they keep positive in titre. And, a linear regression was used to identify the association between maternal antibody level and the time to loss of immunity of neonates. For individuals with right censoring, we treated the time to loss of immunity as missing. On the basis of 225 infants with maternal antibody at birth, time to loss of immunity was defined as the time from baseline to the time when the titre dropped below the cutoff.

At the same time, the survival analysis with considering the interval censor which assuming the outcome occurred over a time interval rather a single time point and the interval regression to estimate the corresponding relationship between the duration and maternal antibody level were conducted as sensitive analyses.

# Supplementary results

# Participant’s characteristics

The characters comparison between participants and local non-participants were presented in table S4. Mothers were similar in terms of age, gravidity, and parity, while the education level for mothers in our study were lower and mothers from Qingtang were relatively overrepresented. Although, 8% of mothers’ blood were collected prior to the delivery, the time of collection was still in the third trimester, when antibody transfer had been completed[9]. And, these disparity of characteristics of mothers was not associated to transfer ratio and did not induce a bias in our study of transfer ratio and maternal titre. For neonates, they were similar in gestational age, sex, birthweight, and the number of twins. The general characters of participants in these two cohorts selected by random sampling were comparable to those of unselected participants, except for selected 1-9 years old children had more older children at baseline (Table S4a, S5).

37% (201/538) of neonates have completed six follow-ups and 71% (1747/2475) children had participated three times annual follow-up visits, they had been allocated to full follow-up group. The baseline characters were similar between full follow-up group and incomplete follow-up group for both neonate cohort and children cohort (Table S4b, S5).

### Table S4 (a). Baseline characteristics of mothers and neonates in neonate cohort between participants and non-participants, selected participants and non-selected participants for CVA16 assay.

|  | **Participants** | **Non-participants** | **Total eligible people in Anhua county** | ***P*_value** |  | **Non-selected objects for CVA16 lab test** | **All objects in neonate cohort** | ***P*_value** |
| --- | --- | --- | --- | --- | --- | --- | --- | --- |
| **Mothers** | **N=534** | **N=2965** | **N=3499** |  |  | **N=520** | **N=1054** |  |
| Age at delivery (years) (n, %) |  |  |  |  |  |  |  |  |
| Median (IQR) | 24 (26-29) | 25 (23-29) | 26 (24-29) | 0.700 |  | 23 (26-29) | 25 (23-29) | 0.713 |
| 14-19 | 18 (3) | 95 (3) | 113 (3) |  |  | 22 (4) | 40 (4) |  |
| 20-29 | 394 (74) | 2096 (72) | 2490 (72) |  |  | 374 (72) | 768 (73) |  |
| 30-48 | 122 (23) | 713 (25) | 835 (24) |  |  | 122 (24) | 244 (23) |  |
| Blood collection time (Range of day) |  |  |  |  |  |  |  |  |
| Before delivery^§^ | 45 (8) | - | - | - |  | 35 (7) | 80 (8) | 0.583 |
| At delivery | 481 (90) | - | - |  |  | 477 (92) | 958 (91) |  |
| After delivery^§^ | 8 (1) | - | - |  |  | 8 (2) | 16 (2) |  |
| Education (n, %) |  |  |  |  |  |  |  |  |
| Middle school or lower | 358 (69) | 2212 (76) | 2570 (75) | <.001 |  | 353 (70) | 711 (70) | 0.434 |
| High school | 144 (28) | 549 (19) | 693 (20) |  |  | 129 (26) | 273 (27) |  |
| University or above | 15 (3) | 135 (5) | 150 (4) |  |  | 21 (4) | 36 (4) |  |
| Township (n, %) |  |  |  |  |  |  |  |  |
| Qingtang  (2·7 people per 10⁴ m²) | 262 (49) | 907 (31) | 1169 (33) | <.001 |  | 224 (43) | 486 (46) | <.001 |
| Jiangnan  (2·1 people per 10⁴ m²) | 159 (30) | 1277 (43) | 1436 (41) |  |  | 223 (43) | 382 (36) |  |
| Tianzhuang  (1·5 people per 10⁴ m²) | 113 (21) | 781 (26) | 894 (26) |  |  | 73 (14) | 186 (18) |  |
| Gravidity (n, %) |  |  |  |  |  |  |  |  |
| 1 | 160 (30) | 837 (29) | 997 (29) | 0.322 |  | 161 (31) | 321 (31) | 0.923 |
| 2 | 221 (42) | 1139 (40) | 1360 (40) |  |  | 210 (41) | 431 (41) |  |
| ≥3 | 149 (28) | 904 (31) | 1053 (31) |  |  | 147 (28) | 296 (28) |  |
| Parity (n, %) |  |  |  |  |  |  |  |  |
| 1 | 248 (47) | 1404 (49) | 1652 (48) | 0.583 |  | 250 (48) | 498 (48) | 0.872 |
| 2 | 265 (50) | 1372 (48) | 1637 (48) |  |  | 253 (49) | 518 (49) |  |
| ≥3 | 17 (3) | 104 (4) | 121 (4) |  |  | 15 (3) | 32 (3) |  |
| **Neonates** | **N=538** | **N=2994** | **N=3532** |  |  | **N=528** | **N=1066** |  |
| Gestational age at birth (weeks), median (IQR) | 40 (39-41) | 40 (39-41) | 40 (39-41) | 0.012^§^ |  | 40 (39-41) | 40 (39-41) | 0.132 |
| Sex (n, %) |  |  |  |  |  |  |  |  |
| Male | 286 (53) | 1478 (52) | 1764 (52) | 0.634 |  | 290 (55) | 576 (54) | 0.606 |
| Female | 252 (47) | 1368 (48) | 1620 (48) |  |  | 238 (45) | 490 (46) |  |
| Birthweight (grams), median (IQR) | 3300 (3000-3600) | 3350 (3000-3600) | 3350 (3000-3600) | 0.241 |  | 3300 (3000-3600) | 3300 (3000-3600) | 0.624 |
| Has twin siblings (n, %) | 12 (2) ^#^ | 54 (2) | 66 (2) | 0.617 |  | 13 (2) | 25 (2)^₤^ | 0.962 |

Date are n (%). ^#^4 pairs of twins, and 4 neonates had a twin sibling but their twin sibling did not participate in CVA16 analysis. ^₤^12 pairs of twins and one single twin sibling were included in whole neonate cohort. Chi-square test and Wilcoxon test were used. ^§^ Significance in statistics test, however, it doesn’t have clinical meaning.

^§^ the blood collection time of these mothers was 1-6 days before or after delivery.

### Table S4 (b). Baseline characteristics of neonates in neonate cohort between full follow-up participants and incomplete follow-up participants.

| **Neonates** | **All participants (N=538)** | | |
| --- | --- | --- | --- |
|  | **Full follow-up (N=201)** | **Incomplete follow-up (N=337)** | ***P*_value** |
| Gestational age at birth (weeks), median (IQR) | 40 (39-41) | 40 (39-41) | 0.606 |
| Sex (n, %) |  |  |  |
| Male | 109 (54) | 177 (53) | 0.768 |
| Female | 92 (46) | 160 (47) |  |
| Birthweight (grams), median (IQR) | 3300 (3000-3600) | 3300 (3000-3600) | 0.991 |
| Has twin siblings (n, %) | 2 (1) | 10 (3) | 0.231 |

| Table S5. Comparison of baseline characteristics of 1-9 years old children between participants and non-selected objects through sampling, full follow-up and incomplete follow-up.  \| **Characteristics** \| **All participants (N=4188)** \| \| \|  \| **Participants with lab test completed (N=2475)** \| \| \| \| --- \| --- \| --- \| --- \| --- \| --- \| --- \| --- \| \| **Lab test completed (n=2475)** \| **Lab test incompleted (n=1713)** \| ***P* value** \|  \| **Full follow up (n=1747)** \| **Incomplete follow up (n=728)** \| ***P* value** \| \| Age at baseline (Year) \|  \|  \|  \|  \|  \|  \|  \| \| Median (IQR) \| 4 (2-6) \| 3 (2-4) \| <0.001 \|  \| 4 (2-6) \| 4 (2-6) \| 0.0743 \| \| 1 \| 355 (14) \| 354 (21) \|  \| 236 (14) \| 119 (16) \| \| 2 \| 368 (15) \| 373 (22) \|  \| 244 (14) \| 124 (17) \| \| 3 \| 332 (13) \| 320 (19) \|  \| 246 (14) \| 86 (12) \| \| 4 \| 333 (13) \| 331 (19) \|  \| 236 (14) \| 97 (13) \| \| 5 \| 330 (13) \| 335 (20) \|  \| 240 (14) \| 90 (12) \| \| 6 \| 283 (11) \| 0 (0) \|  \| 212 (12) \| 71 (10) \| \| 7 \| 205 (8) \| 0 (0) \|  \| 148 (8) \| 57 (8) \| \| 8 \| 162 (7) \| 0 (0) \|  \| 119 (7) \| 43 (6) \| \| 9 \| 107 (4) \| 0 (0) \|  \|  \| 66 (4) \| 41 (6) \|  \| \| Male \| 1246 (50) \| 869 (51) \| 0.8302 \|  \| 874 (50) \| 372 (51) \| 0.6591 \| \| Underlying diseases \| 16 (1) \| 11 (1) \| 1.0000 \|  \| 11 (1) \| 5 (1) \| 1.0000 \| \| Gestational age at birth (weeks) \|  \|  \|  \|  \|  \|  \|  \| \| <37 \| 105 (4) \| 89 (5) \| 0.0553 \|  \| 70 (4) \| 35 (5) \| 0.3310 \| \| 37-42 \| 2344 (95) \| 1616 (94) \|  \| 1661 (95) \| 683 (94) \| \| >42 \| 25 (1) \| 8 (0) \|  \| 15 (1) \| 10 (1) \| \| Delivery mode \|  \|  \|  \|  \|  \|  \|  \| \| Transvaginal delivery \| 1577 (64) \| 1077 (63) \| 0.5875 \|  \| 1106 (63) \| 471 (65) \| 0.5538 \| \| Caesarean \| 897 (36) \| 636 (37) \|  \| 640 (37) \| 257 (35) \| \| Birthweight (grams) \|  \|  \|  \|  \|  \|  \|  \| \| Median (IQR) \| 3250 (3000-3500) \| 3250 (3000-3500) \| 0.4999 \|  \| 3250 (3000-3500) \| 3200 (3000-3500) \| 0.1322 \| \| <2500 \| 88 (4) \| 65 (4) \| 0.8529 \|  \| 63 (4) \| 25 (3) \| 0.8990 \| \| 2500-<4000 \| 2139 (86) \| 1471 (86) \|  \| 1506 (86) \| 633 (87) \| \| ≥4000 \| 247 (10) \| 177 (10) \|  \| 177 (10) \| 70 (10) \| \| Annual family income (RMB, Yuan) \| \|  \|  \|  \|  \|  \|  \| \| <20,000 \| 611 (25) \| 382 (22) \| 0.1070 \|  \| 430 (25) \| 181 (25) \| 0.6055 \| \| 20,000-<50,000 \| 1382 (56) \| 963 (56) \|  \| 985 (56) \| 397 (55) \| \| ≥50,000 \| 482 (19) \| 368 (21) \|  \| 332 (19) \| 150 (21) \| |
| --- | --- | --- | --- | --- | --- | --- | --- | --- | --- | --- | --- | --- | --- | --- | --- | --- | --- | --- | --- | --- | --- | --- | --- | --- | --- | --- | --- | --- | --- | --- | --- | --- | --- | --- | --- | --- | --- | --- | --- | --- | --- | --- | --- | --- | --- | --- | --- | --- | --- | --- | --- | --- | --- | --- | --- | --- | --- | --- | --- | --- | --- | --- | --- | --- | --- | --- | --- | --- | --- | --- | --- | --- | --- | --- | --- | --- | --- | --- | --- | --- | --- | --- | --- | --- | --- | --- | --- | --- | --- | --- | --- | --- | --- | --- | --- | --- | --- | --- | --- | --- | --- | --- | --- | --- | --- | --- | --- | --- | --- | --- | --- | --- | --- | --- | --- | --- | --- | --- | --- | --- | --- | --- | --- | --- | --- | --- | --- | --- | --- | --- | --- | --- | --- | --- | --- | --- | --- | --- | --- | --- | --- | --- | --- | --- | --- | --- | --- | --- | --- | --- | --- | --- | --- | --- | --- | --- | --- | --- | --- | --- | --- | --- | --- | --- | --- | --- | --- | --- | --- | --- | --- | --- | --- | --- | --- | --- | --- | --- | --- | --- | --- | --- | --- | --- | --- | --- | --- | --- | --- | --- | --- | --- | --- | --- | --- | --- | --- | --- | --- | --- | --- | --- | --- | --- | --- | --- | --- | --- | --- | --- | --- | --- | --- | --- | --- | --- | --- |

# Maternal transfer

## 2.1 Maternal antibody titre transfer

| **Index** | **Threshold** | **Mother** | **Neonates’ cord blood** | ***P* value^#^** |
| --- | --- | --- | --- | --- |
| GMT (95% CI) | - | 9.82 (9.14-10.55) | 9.61 (8.97-10.30) | 0.349 |
| Prevalence  (%, 95% CI) | 8 | 44.80 (40.53-49.10) | 41.82 (37.61-46.12) | 0.102 |
|  | 16 | 24.72 (21.13-28.59) | 24.35 (20.78-28.20) | 0.904 |
| Positive participants’ GMT  (95% CI) | 8 | 16.64 (14.92-18.55) | 15.96 (14.38-17.71) | 0.349 |
|  | 16 | 26.80 (23.41-30.67) | 24.60 (21.54-28.07) | 0.216 |

**Table S6. Seroprevalence and GMT of mothers and neonates at birth.**

^#^ Paired t-test or McNemar’s test depended on data.

Only, one pair of mother-neonate has failed to transferring the maternal NT with extremely low transfer ratio (<1/128). For mothers with positive NT, 95% of mother-neonate pairs had a transfer ratio more than 1/4 (Fig. S3).

~~
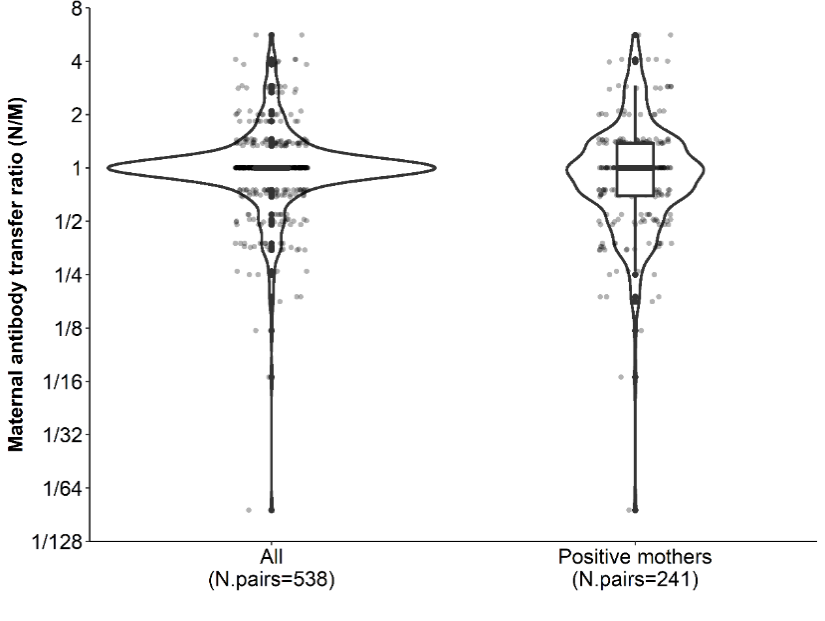
~~

### Fig. S3. Distribution of maternal antibody titre transfer ratio against CVA16 for all pairs of neonate-mother (A) and positive mothers with antibody titre ≥8 (B).


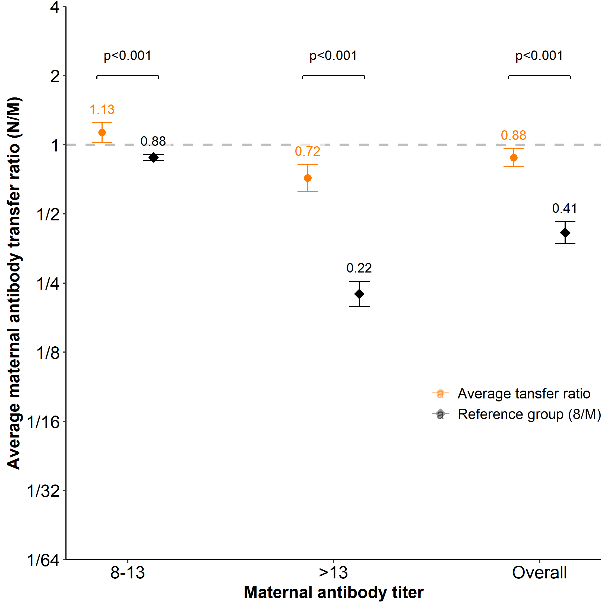


### Fig. S4. The aggregated maternal titre specified transfer ratio.


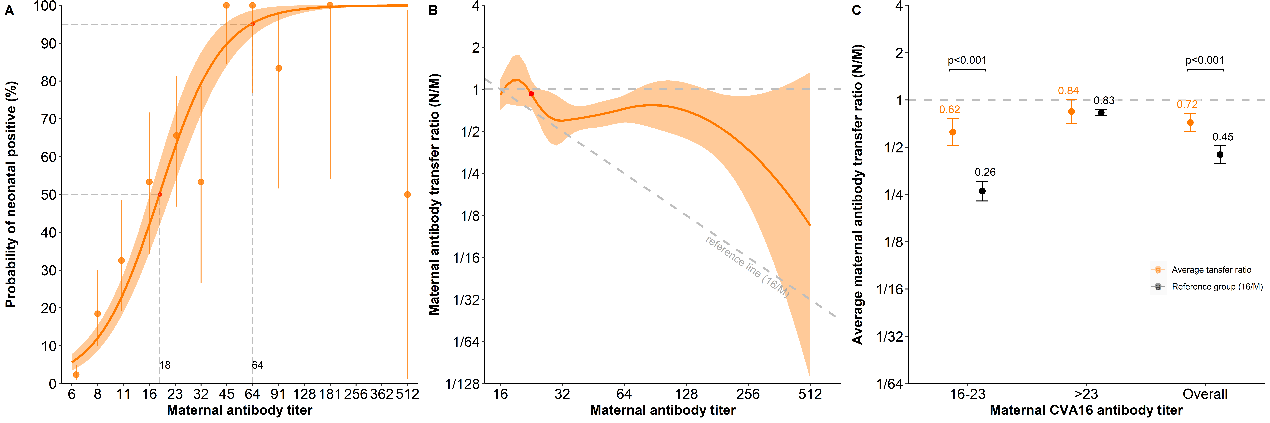


### Fig. S5. Maternal antibody titre transfers efficacy against CVA16. The probability for neonatal positivity over different maternal antibody level (A) and the transfer ratio trend over maternal antibody titre (B) and the aggregated maternal titre specified transfer ratio (C). Grey dashed lines in panel B, and C represented 100% seroprevalence with 16 as threshold, neonates got the titre ≥ 16, and the same transfer ratio, respectively. One side paired t test was used to test the difference between observed transfer ratio and the reference group.

### Table S7. Sample size (n, %) for CVA16 antibody transfer ratio analysis.

| **Positive**  **antibody threshold** | **Maternal titre** | **Neonatal titre** | | | | **Baseline analysis** | **Sensitive analysis with total pairs of mother-neonate** |
| --- | --- | --- | --- | --- | --- | --- | --- |
| 8 |  | <8 | | | ≥8 |  |  |
|  | <8 | 263 (49) | | | 34 (6) | 241 (44) | 538 (100) |
|  | ≥8 | 50 (9) | | | 191 (36) |  |  |
| 16 |  | | <16 | ≥16 | |  |  |
|  | <16 | 372 (69) | | | 33 (6) | 133 (25) | 538 (100) |
|  | ≥16 | 35 (7) | | | 98 (18) |  |  |

Using the total pairs of mother-neonate, the overall maternal CVA16 antibody titre transfer ratio is 0.98 (95% CI: 0.94, 1.02), while, it was significantly decreased with maternal antibody titre increase, which the average transfer ratios for mothers were more than 1 when maternal antibody titre ≤13 (Fig. S6). Around 10% of neonates are positive in contract to the negative mothers, which may be in results of the false negative or false positive of both NT assay. Another reason may be truth in individual level.

**
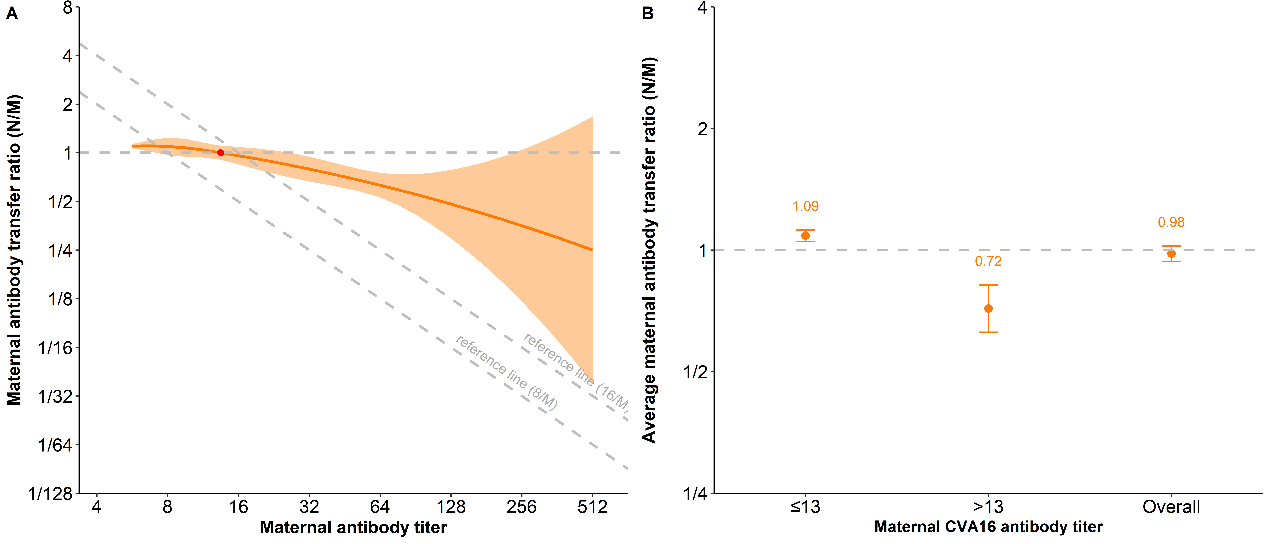
**

### Fig. S6. Transfer ratio trend with all pairs of mother-neonate.

## 2.2 Maternal transfer ratio associated factors

### Table S8. Multivariate analysis with factors associated with neonates’ seropositivity, antibody titre at birth and transfer ratio.

| **Characteristics** | **β (95% CI)** | **2^β^ (95% CI)** | ***P value*** |
| --- | --- | --- | --- |
| **Factors associated with seropositivity in neonates** | | | |
| Log (Maternal titre) | 0.235 (0.208, 0.263) | 1.177 (1.155, 1.200) | <0.001 |
| Twin |  |  |  |
| Yes | -0.246 (-0.473, -0.020) | 0.843 (0.720, 0.986) | 0.034 |
| Maternal age at delivery (Years) | -0.005 (-0.012, 0.002) | 0.997 (0.992, 1.001) | 0.131 |
| Interval between sampling and delivery of mother | |  |  |
| At delivery | Reference | Reference |  |
| Before delivery | -0.111 (-0.231, 0.009) | 0.926 (0.852, 1.006) | 0.070 |
| After delivery | -0.106 (-0.382, 0.171) | 0.929 (0.767, 1.126) | 0.454 |
| **Factors associated with log-transformed titre of anti-CVA16 antibodies in neonates** | | | |
| Log (Maternal titre) | 0.758 (0.708, 0.809) | 1.691 (1.634, 1.752) | <0.001 |
| Maternal age at delivery (Years) | -0.012 (-0.024, 0.001) | 0.992 (0.984, 1.001) | 0.069 |
| Twin |  |  |  |
| Yes | -0.167 (-0.976, 0.642) | 0.891 (0.508, 1.560) | 0.686 |
| Education |  |  |  |
| Middle school or lower | Reference | Reference |  |
| High school | -0.024 (-0.164, 0.117) | 0.984 (0.893, 1.084) | 0.742 |
| University or above | -0.039 (-0.411, 0.333) | 0.973 (0.752, 1.260) | 0.838 |
| Annual family income (CNY) |  |  |  |
| <20,000 | Reference | Reference |  |
| [20,000,50,000] | -0.052 (-0.229, 0.124) | 0.965 (0.853, 1.090) | 0.563 |
| ≥50,000 | -0.074 (-0.267, 0.119) | 0.950 (0.831, 1.086) | 0.454 |
| **Factors associated with transfer ratio of maternal anti-CVA16 antibodies** | | | |
| Log (Maternal titre) | -0.231 (-0.280, -0.181) | 0.852 (0.824, 0.882) | <0.001 |
| Mather with liver system disease  (Hepatitis B virus carriers or chronic hepatitis B) | |  |  |
| Yes | -0.258 (-0.475, -0.041) | 0.836 (0.719, 0.972) | 0.020 |
| Parity |  |  |  |
| 2 | Reference | Reference |  |
| 1 | 0.120 (-0.003, 0.243) | 1.087 (0.998, 1.183) | 0.056 |
| ≥3 | 0.044 (-0.301, 0.388) | 1.031 (0.812, 1.309) | 0.804 |

Antibody titres were log2-transformed in linear regression. β>0 indicates that the predictors were associated with increases of anti-CVA16 antibodies by (2^β^–1) folds; β<0 indicates the predictors were associated with decreases of anti-CVA16 antibodies by (1–2^β^) folds. While for logistic regression to analysis the factors associated with seropositivity in neonates, β indicates the probability of seropositive was exp (β) comparing to seronegative, namely odds ratio (OR).

### Table S9. Univariate analysis with factors associated with neonates’ seropositivity at birth.

|  | **Seropositive** | | **OR (95% CI)** | ***P* value** |
| --- | --- | --- | --- | --- |
|  | **No** | **Yes** |  |  |
| Log (Maternal titre) (mean ± sd) | |  |  |  |
|  | 2.68 ± 0.60 | 4.15 ± 1.35 | 6.67 (4.75, 9.77) | <0.001 |
| Maternal age (years) (mean ± sd) | |  |  |  |
|  | 26.92 ± 5.07 | 25.88 ± 4.66 | 0.96 (0.92, 0.99) | 0.016 |
| Birthweight (g) (mean ± sd) | |  |  |  |
|  | 3326.49 ± 461.15 | 3306.89 ± 404.37 | 1.00 (1.00, 1.00) | 0.609 |
| Gestational age (weeks) (mean ± sd) | |  |  |  |
|  | 40.02 ± 1.42 | 39.91 ± 1.53 | 0.95 (0.84, 1.07) | 0.384 |
| Sex |  |  |  |  |
| Male | 169 (54) | 117 (52) | Reference |  |
| Female | 144 (46) | 108 (48) | 1.08 (0.77, 1.53) | 0.648 |
| Twin |  |  |  |  |
| No | 302 (96) | 224 (100) | Reference |  |
| Yes | 11 (4) | 1 (0) | 0.12 (0.01, 0.64) | 0.045 |
| Time of maternal blood collection | |  |  |  |
| At delivery | 275 (88) | 209 (93) | Reference |  |
| Before delivery | 32 (10) | 14 (6) | 0.58 (0.29, 1.09) | 0.098 |
| After delivery | 6 (2) | 2 (1) | 0.44 (0.06, 1.93) | 0.316 |
| Gravidity |  |  |  |  |
| 2 | 123 (39) | 101 (45) | Reference |  |
| 1 | 97 (31) | 64 (29) | 0.80 (0.53, 1.21) | 0.297 |
| ≥3 | 92 (29) | 58 (26) | 0.77 (0.50, 1.17) | 0.219 |
| Parity |  |  |  |  |
| 2 | 151 (48) | 100 (45) | Reference |  |
| 1 | 149 (48) | 118 (53) | 1.20 (0.84, 1.70) | 0.316 |
| ≥3 | 12 (4) | 5 (2) | 0.63 (0.20, 1.75) | 0.398 |
| Town |  |  |  |  |
| Qingtang | 154 (49) | 110 (49) | Reference |  |
| Jiangnan | 95 (30) | 65 (29) | 0.96 (0.64, 1.43) | 0.833 |
| Tianzhuang | 64 (20) | 50 (22) | 1.09 (0.70, 1.70) | 0.692 |
| Education |  |  |  |  |
| Middle school or lower | 200 (66) | 158 (73) | Reference |  |
| High school | 91 (30) | 53 (25) | 0.74 (0.49, 1.09) | 0.133 |
| University or above | 10 (3) | 5 (2) | 0.63 (0.19, 1.82) | 0.412 |
| Annual family income (CNY) | |  |  |  |
| <20,000 | 44 (14) | 38 (17) | Reference |  |
| [20,000,50,000) | 173 (55) | 124 (55) | 0.83 (0.51, 1.36) | 0.457 |
| ≥50,000 | 95 (30) | 63 (28) | 0.77 (0.45, 1.32) | 0.336 |
| Mode of delivery |  |  |  |  |
| Transvaginal delivery | 188 (60) | 143 (64) | Reference |  |
| Caesarean section | 125 (40) | 82 (36) | 0.86 (0.60, 1.23) | 0.412 |
| Mather with liver system disease (Hepatitis B virus carriers or chronic hepatitis B) | | | |  |
| No | 277 (83) | 206 (87) | Reference |  |
| Yes | 29 (17) | 15 (13) | 0.70 (0.35, 1.31) | 0.273 |
| Gestational diabetes |  |  |  |  |
| No | 303 (100) | 220 (100) | - | - |
| Yes | 1 (0) | 0 (0) | - | - |
| Gestational hypertension | |  |  |  |
| No | 301 (99) | 219 (100) | Reference |  |
| Yes | 3 (1) | 1 (0) | 0.46 (0.02, 3.61) | 0.500 |

### Table S10. Univariate analysis with factors associated with the transfer ratio of maternal antibody.

| **Characteristics** | **β (95% CI)** | **2^β^ (95% CI)** | ***P* value** |
| --- | --- | --- | --- |
| Log (Maternal titre) |  |  |  |
|  | -0.23 (-0.28, -0.18) | 0.85 (0.82, 0.88) | <0.001 |
| Maternal age (years) |  |  |  |
|  | -0.01 (-0.02, 0.01) | 1.00 (0.99, 1.00) | 0.291 |
| Birthweight (g) |  |  |  |
|  | 0.00 (0.00, 0.00) | 1.00 (1.00, 1.00) | 0.175 |
| Gestational age (weeks) |  |  |  |
|  | 0.00 (-0.05, 0.04) | 1.00 (0.97, 1.03) | 0.844 |
| Neonate sex |  |  |  |
| Female | 0.03 (-0.10, 0.16) | 1.02 (0.93, 1.12) | 0.631 |
| Twin |  |  |  |
| Yes | -0.31 (-0.74, 0.13) | 0.81 (0.60, 1.09) | 0.163 |
| Interval between sampling and delivery of mother | |  |  |
| At delivery | Reference | Reference |  |
| Before delivery | -0.13 (-0.36, 0.10) | 0.91 (0.78, 1.07) | 0.258 |
| After delivery | 0.02 (-0.51, 0.55) | 1.01 (0.70, 1.47) | 0.942 |
| Gravidity |  |  |  |
| 2 | Reference | Reference |  |
| 1 | 0.06 (-0.09, 0.21) | 1.04 (0.94, 1.16) | 0.444 |
| ≥3 | -0.06 (-0.22, 0.09) | 0.96 (0.86, 1.07) | 0.423 |
| Parity |  |  |  |
| 2 | Reference | Reference |  |
| 1 | -0.17 (-0.30, -0.04) | 0.89 (0.81, 0.97) | 0.012 |
| ≥3 | -0.09 (-0.46, 0.30) | 0.94 (0.73, 1.22) | 0.646 |
| Town |  |  |  |
| Qingtang | Reference | Reference |  |
| Jiangnan | 0.04 (-0.11, 0.19) | 1.03 (0.93, 1.14) | 0.618 |
| Tianzhuang | 0.03 (-0.14, 0.19) | 1.02 (0.91, 1.14) | 0.766 |
| Education |  |  |  |
| Middle school or lower | Reference | Reference |  |
| High school | 0.05 (-0.10, 0.20) | 1.04 (0.94, 1.15) | 0.494 |
| University or above | 0.08 (-0.32, 0.47) | 1.06 (0.80, 1.39) | 0.699 |
| Annual family income (CNY) |  |  |  |
| <20,000 | Reference | Reference |  |
| [20,000,50,000) | -0.03 (-0.22, 0.15) | 0.98 (0.86, 1.11) | 0.713 |
| ≥50,000 | 0.00 (-0.20, 0.21) | 1.00 (0.87, 1.15) | 0.979 |
| Mode of delivery |  |  |  |
| Transvaginal delivery | Reference | Reference |  |
| Caesarean section | -0.09 (-0.22, 0.04) | 0.94 (0.86, 1.03) | 0.193 |
| Mather with liver system disease (Hepatitis B virus carriers or chronic hepatitis B) | | |  |
| No | Reference | Reference |  |
| Yes | -0.27 (-0.50, -0.032) | 0.83 (0.71, 0.98) | 0.026 |
| Gestational diabetes |  |  |  |
| No | Reference | Reference |  |
| Yes | -0.47 (-1.97, 1.02) | 0.72 (0.26, 2.03) | 0.534 |
| Gestational hypertension |  |  |  |
| No | Reference | Reference |  |
| Yes | 0.28 (-0.47, 1.03) | 1.21 (0.72, 2.04) | 0.466 |

Antibody titres were log2-transformed in linear regression. β>0 indicates that the predictors were associated with increases of anti-CVA16 antibodies by (2^β^–1) folds; β<0 indicates the predictors were associated with decreases of anti-CVA16 antibodies by (1–2^β^) folds.

###

### Table S11. Univariate analysis with factors associated with neonates’ antibody titre at birth.

| **Characteristics** | **β (95% CI)** | **2^β^ (95% CI)** | ***P* value** |
| --- | --- | --- | --- |
| Log (Maternal titre) |  |  |  |
|  | 0.77 (0.72, 0.82) | 1.70 (1.65, 1.76) | <0.001 |
| Maternal age at delivery (years) |  |  |  |
|  | -0.03 (-0.05, -0.01) | 0.98 (0.97, 0.99) | 0.003 |
| Birthweight (g) |  |  |  |
|  | 0 (-0.00, 0.00) | 1.00 (1.00, 1.00) | 0.966 |
| Gestational age (weeks) |  |  |  |
|  | -0.02 (-0.09, 0.05) | 0.98 (0.94, 1.03) | 0.517 |
| Neonate sex |  |  |  |
| female | 0.10 (-0.10, 0.30) | 1.07 (0.94, 1.23) | 0.312 |
| Twin |  |  |  |
| Yes | -0.70 (-1.37, -0.03) | 0.62 (0.39, 0.98) | 0.042 |
| Time of maternal blood collection |  |  |  |
| At delivery | Reference | Reference |  |
| Before delivery | -0.19 (-0.54, 0.17) | 0.88 (0.69, 1.12) | 0.299 |
| After delivery | -0.35 (-1.17, 0.47) | 0.79 (0.44, 1.39) | 0.405 |
| Gravidity |  |  |  |
| 2 | Reference | Reference |  |
| 1 | -0.08 (-0.32, 0.16) | 0.94 (0.80, 1.11) | 0.489 |
| ≥3 | -0.15 (-0.39, 0.10) | 0.90 (0.76, 1.07) | 0.240 |
| Parity |  |  |  |
| 2 | Reference | Reference |  |
| 1 | 0.06 (-0.14, 0.26) | 1.04 (0.90, 1.20) | 0.571 |
| ≥3 | -0.06 (-0.64, 0.52) | 0.96 (0.64, 1.43) | 0.832 |
| Town |  |  |  |
| Qingtang | Reference | Reference |  |
| Jiangnan | -0.18 (-0.41, 0.05) | 0.88 (0.75, 1.03) | 0.120 |
| Tianzhuang | -0.14 (-0.40, 0.12) | 0.91 (0.76, 1.09) | 0.294 |
| Education |  |  |  |
| Middle school or lower | Reference | Reference |  |
| High school | -0.19 (-0.42, 0.04) | 0.88 (0.75, 1.03) | 0.099 |
| University or above | -0.30 (-0.91, 0.31) | 0.81 (0.53, 1.24) | 0.338 |
| Annual family income (CNY) |  |  |  |
| <20,000 | Reference | Reference |  |
| [20,000,50,000) | -0.10 (-0.38, 0.19) | 0.94 (0.77, 1.14) | 0.516 |
| >=50,000 | -0.26 (-0.58, 0.05) | 0.83 (0.67, 1.04) | 0.099 |
| Mode of delivery |  |  |  |
| Transvaginal delivery | Reference | Reference |  |
| Caesarean section | -0.13 (-0.33, 0.08) | 0.91 (0.79, 1.05) | 0.218 |
| Mather with liver system disease (Hepatitis B virus carriers or chronic hepatitis B) | | |  |
| No | Reference | Reference |  |
| Yes | -0.22 (-0.58, 0.14) | 0.86 (0.67, 1.11) | 0.237 |
| Gestational diabetes |  |  |  |
| No | Reference | Reference |  |
| Yes | -0.77 (-3.08, 1.54) | 0.59 (0.12, 2.91) | 0.513 |
| Gestational hypertension |  |  |  |
| No | Reference | Reference |  |
| Yes | -0.02 (-1.18, 1.14) | 0.99 (0.44, 2.20) | 0.974 |

Antibody titres were log2-transformed in linear regression. β>0 indicates that the predictors were associated with increases of anti-CVA16 antibodies by (2^β^–1) folds; β<0 indicates the predictors were associated with decreases of anti-CVA16 antibodies by (1–2^β^) folds.

### Table S12. Sensitive analysis for multivariate analysis with factors associated with maternal antibody transfer ratio.

| **Characteristics** | **β (95% CI)** | **2^β^ (95% CI)** | ***P* value** |
| --- | --- | --- | --- |
| **Model 1** | | | |
| Log (Maternal titre) | -0.288 (-0.385, -0.191) | 0.819 (0.766, 0.876) | <0.001 |
| Mather with liver system disease (Hepatitis B virus carriers or chronic hepatitis B) | | |  |
| Yes | -0.484 (-0.941, -0.026) | 0.715 (0.521, 0.982) | 0.039 |
| Twin |  |  |  |
| Yes | -1.559 (-3.456, 0.338) | 0.339 (0.091, 1.264) | 0.109 |
| Parity |  |  |  |
| 2 | Reference | Reference |  |
| 1 | 0.256 (0.003, 0.509) | 1.194 (1.002, 1.423) | 0.049 |
| ≥3 | 0.418 (-0.378, 1.215) | 1.336 (0.770, 2.321) | 0.304 |
| **Model 2** | | | |
| Log (Maternal titre) | -0.356 (-0.441, -0.272) | 0.781 (0.737, 0.828) | <0.001 |
| Mather with liver system disease (Hepatitis B virus carriers or chronic hepatitis B) | | |  |
| Yes | -0.477 (-0.908, -0.045) | 0.718 (0.533, 0.969) | 0.031 |
| Twin |  |  |  |
| Yes | -1.625 (-3.461, 0.212) | 0.324 (0.091, 1.158) | 0.084 |
| Parity |  |  |  |
| 2 | Reference | Reference |  |
| 1 | 0.227 (-0.001, 0.456) | 1.170 (0.999, 1.372) | 0.052 |
| ≥3 | 0.356 (-0.413, 1.126) | 1.280 (0.751, 2.183) | 0.365 |

# 0-13 years old children CVA16 antibody dynamic

The sensitive analysis with 16 as positive threshold shows that the proportion of positive maternally transmitted antibodies acquired by newborns at birth is very low, and the average level of the population has reached the positive level until 2.7 (range: 2.4-3.1) years old (Fig. S7).


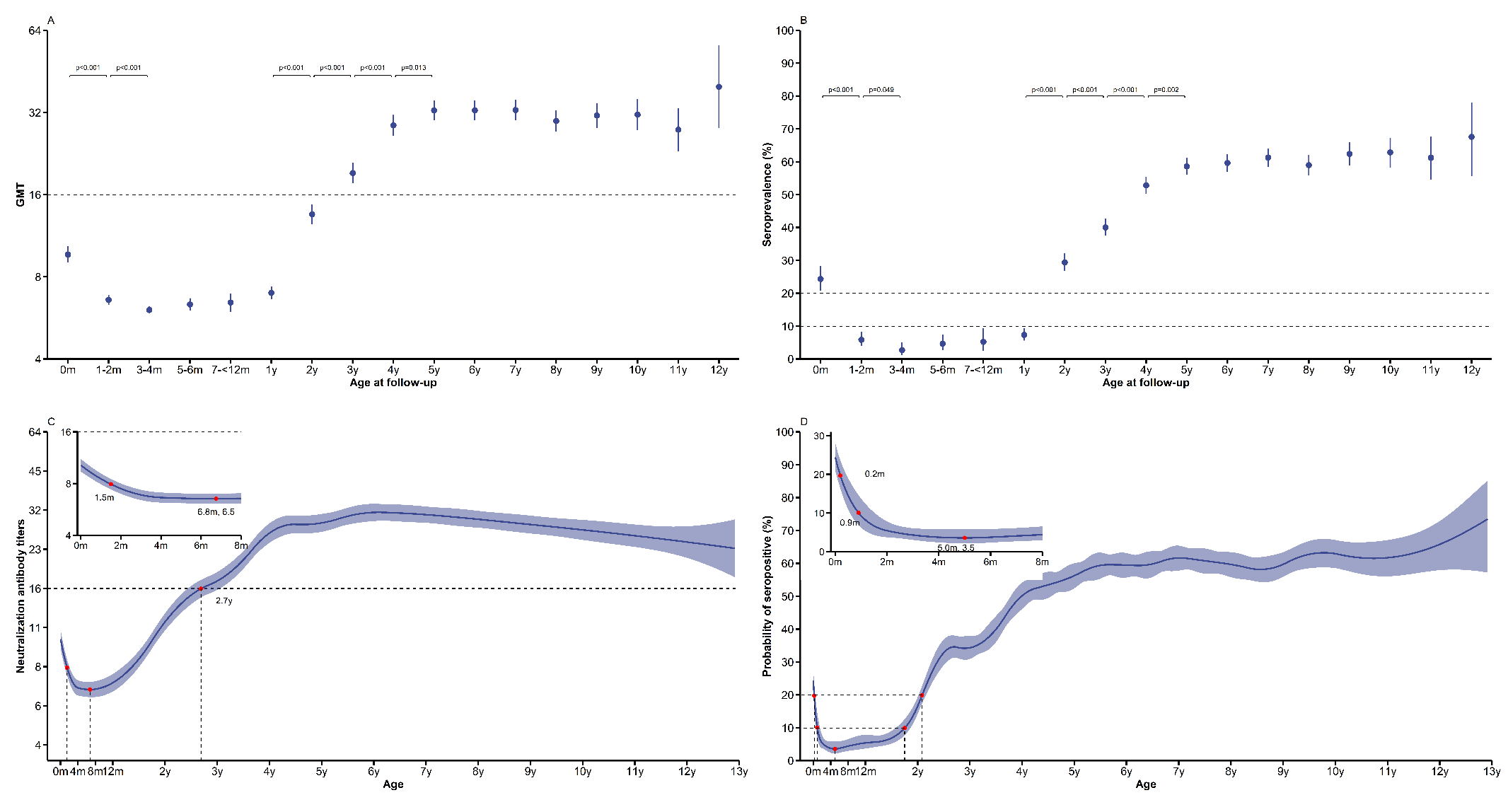


### Fig. S7. Dynamic of seroprevalence, neutralization antibody titre by age for neonates using 16 as positive threshold.


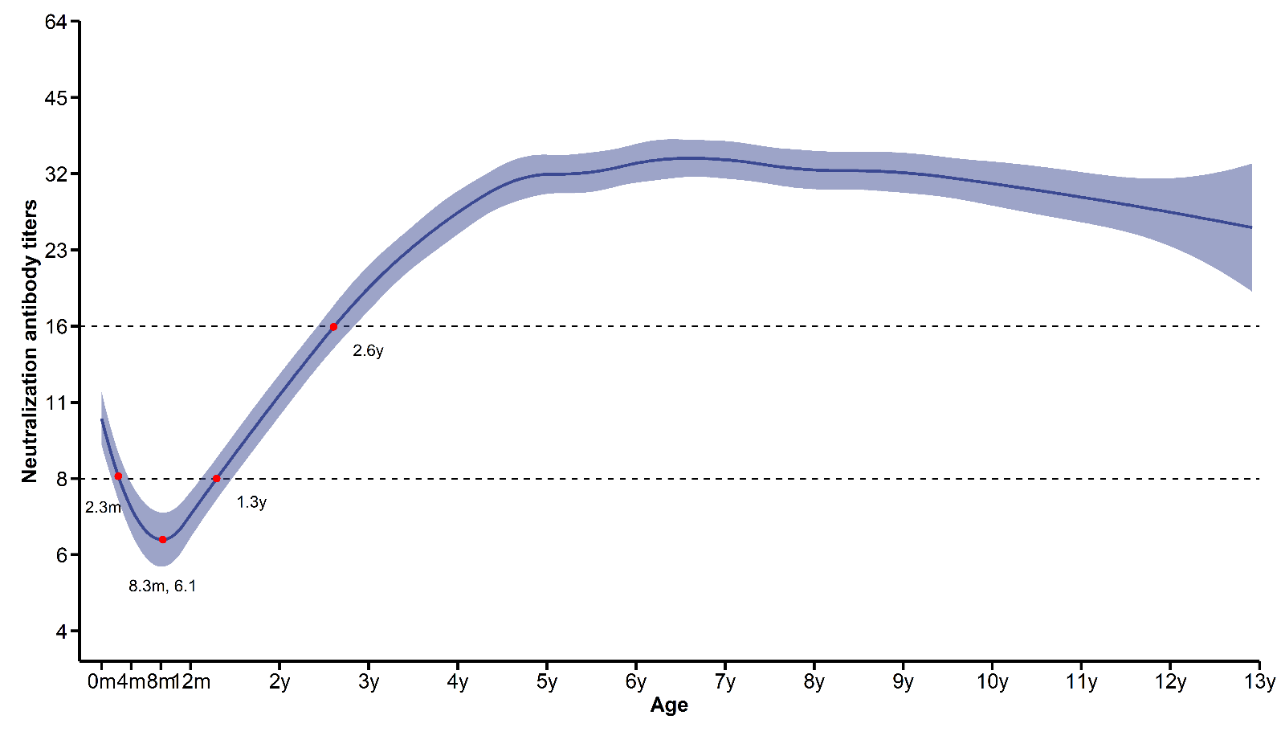
A sensitive analysis was conducted to estimate the impact of losing follow-up (table S4b, Fig. S8), choosing a subset of participants with 201 neonates and 4174 children aged 1-9 years at baseline who participated all follow-up. The overall GMT decayed to under 8 at 2.3 (range: 1.3-3.8) months, then increased to 8 and 16 at 1.3 (range: 1.1-1.5) and 2.6 (range: 2.4-2.8) years old, respectively.

### Fig. S8. Dynamic of neutralization antibody titre by age for neonates using full follow-up subset.

To explore the effect of maternal antibody level on the children’s antibody dynamics, using a neonate cohort, we aggregated the children according to maternal antibody titre into three groups (negative group: <8, low level: 8-<64, and high level: 64-512), with the range of maternal antibody being 6 to 512. After that, we performed a subgroup analysis to analyse whether maternal antibody levels had an effect on antibody dynamics, and the results are listed as follows:

Similar to the overall dynamics of all participants (Fig. 3A), the antibody titers from the two positive groups both decreased from birth to the bottom and then rebounded due to natural infection. In contrast, for the negative group, the antibody titer was always under the detectable level at birth and then increased due to natural infection. There was a significant difference in the antibody dynamics within 6 months between these three groups. However, the difference in antibody levels was not obvious when the patient was more than 1.5 years old (Fig. S9).


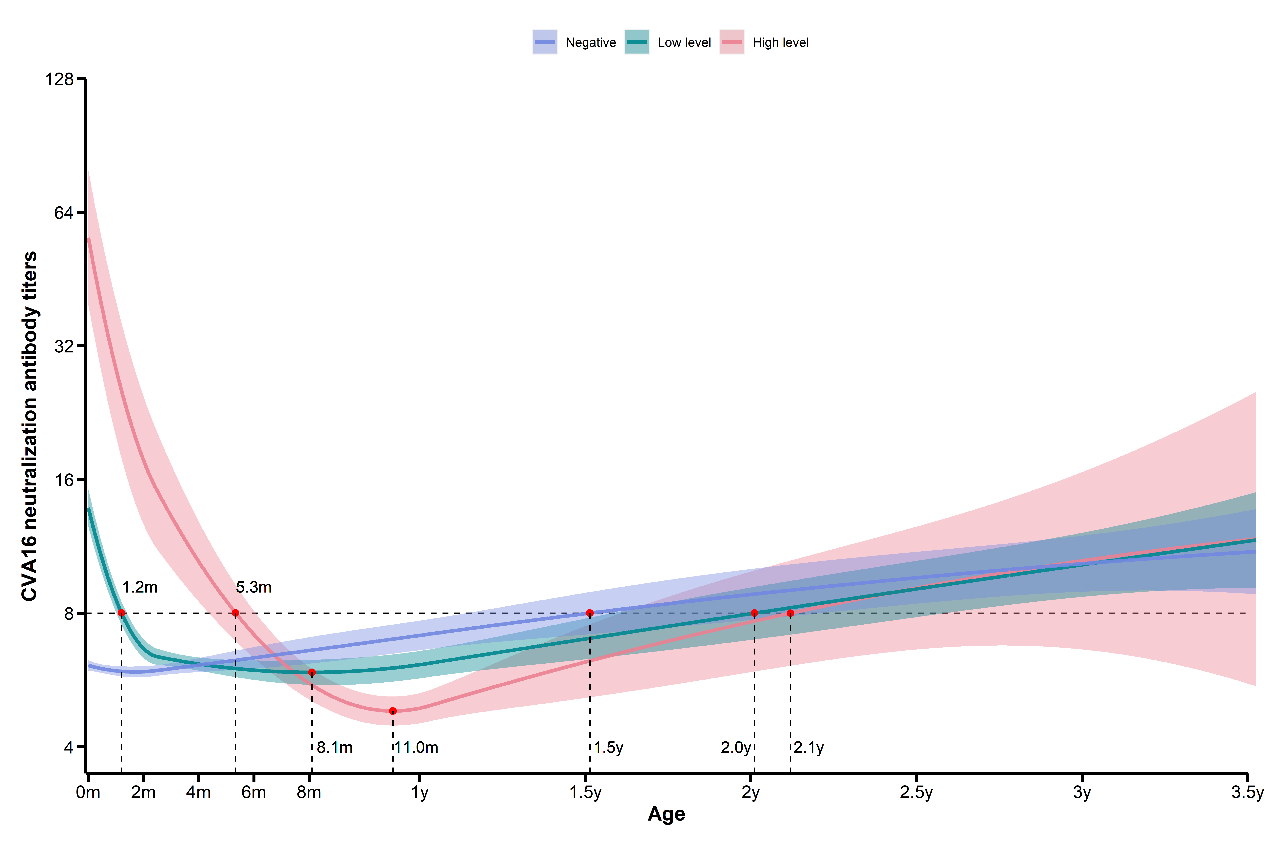


### Fig. S9. Dynamics of neutralization antibody titer by age for neonates stratified by maternal antibody levels.

# Decay rate/half-life and time to reduction to negative

On average, CVA16 maternal successfully transmitted antibody had decay under negative around 84 days (95% CI 75-94). While, due to the relative high-level GMT induced by natural infection and slower decay rate, the natural infection induced antibody did not decay to negative until 40 months. Besides that, age at infection plays no effect on the decay rate of natural infected antibody (*p* = 0.08).


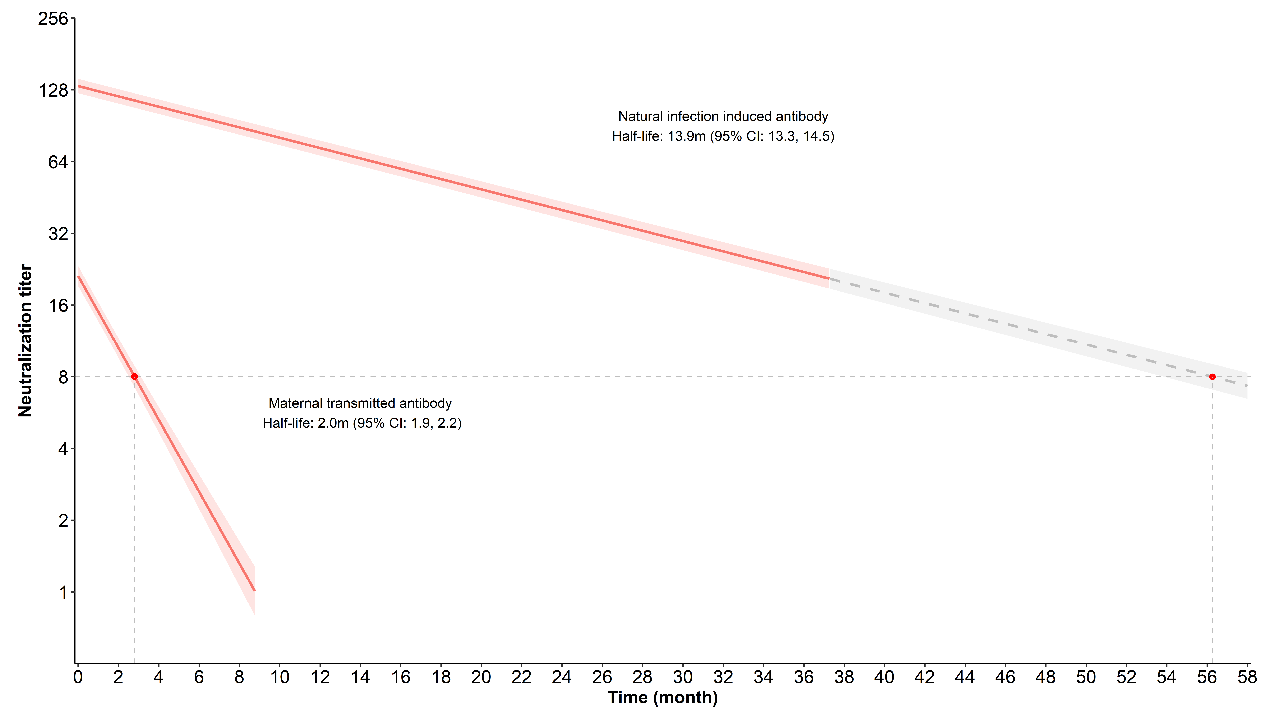


### Fig. S10: Waning rate of maternal transmitted CVA16 antibody and natural infection induced antibody.

### Table S13. Sensitive analysis of median age of neonate with positive antibody.

| Number | Positive threshold | No. of neonates with positive antibody titre | Method of estimation | Median time for losing positive antibody titre (mean, 95% CI) | Prolong time of losing positive immunity with maternal titre increasing 2-fold (mean, 95% CI) |
| --- | --- | --- | --- | --- | --- |
| Baseline analysis | 8 | 225 | Right censor | 2.43 (2.33, 2.53) | 38 (28, 48) |
| Sensitive analysis | 8 | 225 | Interval censor | 1.95 (0.92 4.02) | 19 (16, 23) |
|  | 16 | 131 | Right censor | 2.33 (2.30, 2.40) | 25 (14, 36) |
|  | 16 | 131 | Interval censor | 1.95 (1.92, 2.68) | 16 (12, 20) |

Neonatal antibody level has no significant effect on half-life and time to loss protection.

# Disease burden of HFMD related to CVA16

The 1-year-old group was also the age group accounted for the largest number of severe and mild symptomatic HFMD cases related to CVA16 in Hunan province with 50.8%, 30.4%, respectively (Fig. S11).


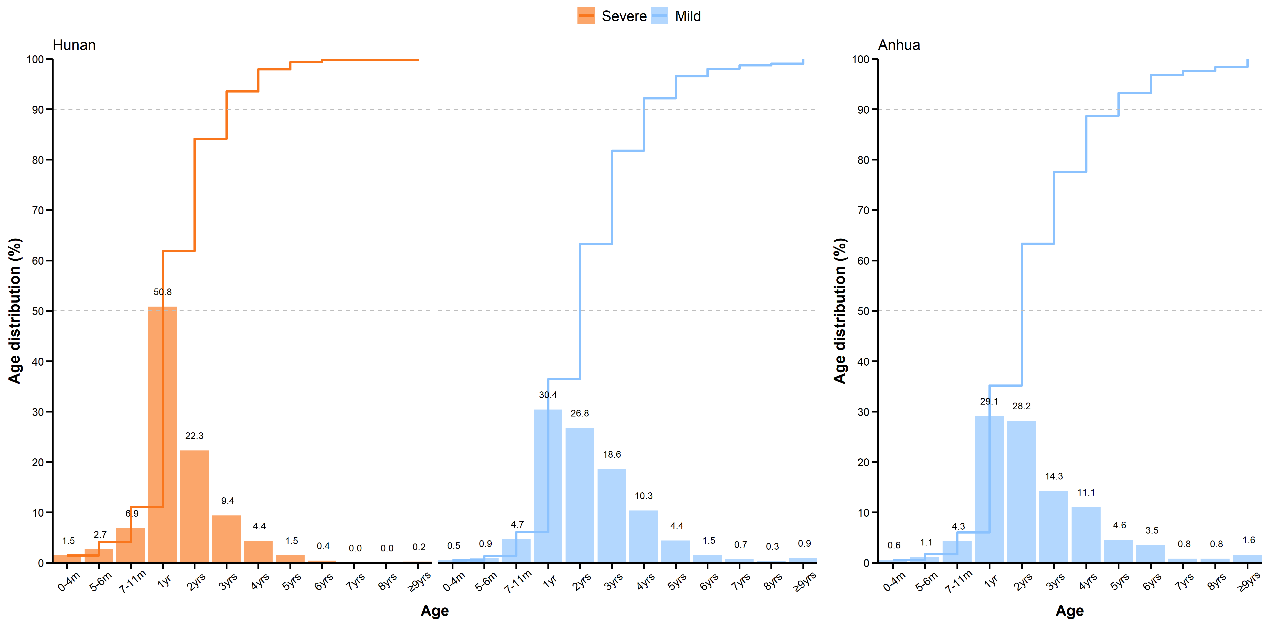


### Fig. S11. Age distribution and cumulative distribution of HFMD associated to CVA16 by clinical severity in Hunan province during 2009-2018 and Anhua county during 2013-2016.
